# Shotgun Glycomics Identifies Tumor-Associated Glycan Ligands Bound by an Ovarian Carcinoma-Specific Monoclonal Antibody

**DOI:** 10.1038/s41598-017-15123-z

**Published:** 2017-11-03

**Authors:** B. Liau, B. Tan, G. Teo, P. Zhang, A. Choo, P. M. Rudd

**Affiliations:** Analytics Department, Bioprocessing Technology Institute, Agency for Science, Technology and Research, 20 Biopolis Way, Singapore, 138668 Republic of Singapore

## Abstract

Cancers display distinctive carbohydrate molecules (glycans) on their surface proteins and lipids. mAb A4, an in-house generated monoclonal IgM antibody, is capable of distinguishing malignant ovarian carcinoma cells from benign ovarian epithelia by binding specifically to cancer cell-associated glycans. However, the structural details of the glycan targets of mAb A4 have been elusive. Here we developed a novel approach of isolating and fractionating glycan molecules released from glycoproteins in cancer cell lysates using HILIC-UPLC, and used them as probes on a microarray for affinity-based identification of the binding targets, allowing full-size, difficult to synthesize, cancer-associated glycans to be directly studied. As a result of this “shotgun” glycomics approach, we corroborate the previously assigned specificity of mAb A4 by showing that mAb A4 binds primarily to large (>15 glucose units), sialylated N-glycans containing the H-type 1 antigen (Fuc-α1,2-Gal-β1,3-GlcNAc). Although mAb A4 was also capable of directly binding to type 1 N-acetyl-lactosamine, this epitope was mostly shielded by sialylation and thus relatively inaccessible to binding. Knowledge of the structure of mAb A4 antigen will facilitate its clinical development as well as its use as a diagnostic biomarker.

## Introduction

Cancer cells have long been known to harbour distinctive glycan structures on their cell surface and secreted glycoproteins, such as increased core and outer arm fucosylation^1–5^, increased Lewis antigen expression^5–7^, increased sialylation^3,8,9^, and truncation of O-glycans to T and Tn antigens, as well as their sialylated counterparts^9–12^. Monoclonal antibodies that bind to a subset of cancer cell-associated glycans can be generated using classical hybridoma-based techniques^13–16^, and represent promising new drug candidates that may have a role to play in the generation of new immune-therapies such as chimeric antigen receptor T (CAR-T) cells^17,18^. One such antibody, mAb A4, was recently produced in the lab of Dr. Andre Choo, and has been shown to be capable of distinguishing breast, ovary, testis, lung, pancreas, bone and small intestine cancer cells from their benign counterparts in immuno-histochemical experiments^19^. Although the specificity of mAb A4 was successfully characterized by Choo *et al*. using a combination of mass spectrometry and siRNA-based approaches^19^, the fact remains that identifying the antigen targets of this promising class of therapeutic molecules often requires bespoke methods and would benefit from an easily scalable approach that could be applied in parallel to many antibodies. Towards this end, we have applied the technique of “shotgun” glycan microarrays pioneered by the Cummings group^20–22^, as an alternative method to elucidate the glycan antigens bound by mAb A4.

“Shotgun” glycan microarrays have proven to be a powerful technique for analyzing protein-carbohydrate binding interactions. To date, they have been used to identify biologically relevant brain glycosphingolipids^20^, pig lung viral receptors^21^, Schistosoma Mansoni surface glycans^22^ and milk oligosaccharides that are potential decoy receptors for rotaviruses^23^. Their power comes from the fact that glycan probes are purified directly from the biological context of interest – as such, this approach is not biased by the experimenter’s choice of which glycans to include on the array, and is also not subject to the limitations of chemical or chemo-enzymatic synthesis, which remains a challenging proposition and an active area of research^24–26^. Shotgun glycan microarrays therefore contain all the complexity needed to study the problem at hand. In contrast, chemically defined glycan arrays contain only a subset of the structures relevant to a particular biological context and may therefore fail to identify all biologically relevant ligands^27,28^. Moreover, they typically provide information only about the minimal structures necessary for binding, providing little information about the rest of the glycan.

One of the main drawbacks of the shotgun glycomics approach is that it currently requires many sample handling steps since multi-dimensional chromatography is used to separate glycans from complex mixtures. In practice, the technique is therefore limited to biological samples that are relatively glycan-rich to compensate for the inevitable sample loss. This has made it difficult to apply to cancer cells or cell lines, because they typically yield only small quantities of glycans. For this reason, researchers typically rely on a source of glycans other than cancer cell lines, such as porcine stomach mucin^27,28^, when fabricating natural glycan arrays to study cancer-specific antibodies. Whilst this approach has been successful in identifying binding epitopes, the findings may not be wholly applicable to cancer biology because the glycans were not drawn from cancer cells. Questions arise as to whether the identified epitopes were present on cancer cells, and if so what the full structures of the epitope-containing glycans were in a cancer context.

In this study, we have modified the traditional approach to shotgun glycomics^20–22^ by relying on a single HILIC-UPLC separation step instead of two-dimensional separation with a normal phase chromatography step followed by Porous Graphitic Carbon (PGC). HILIC is an extension of normal phase chromatography, and relies on interactions between glycans and hydrophilic chemical groups presented on the stationary phase under high concentrations of organic solvent to affect the separation of glycan mixtures. HILIC has a number of advantages over PGC: firstly, the order of elution with HILIC is highly predictable because retention times strongly correlate with the size of the glycan, whereas elution order on PGC is less predictable^29^. Secondly, PGC is strongly retentive and thus may not elute highly sialylated glycans completely, moreover it is prone to fouling, causing retention time drift^30^. The use of a single separation step greatly reduced the number of sample handling steps, and allowed us to directly use cancer cell line glycans to fabricate shotgun glycan arrays despite their paucity, instead of relying on surrogate materials such as porcine intestinal lining^31^. Cancer cell line glycans were then sequenced on-chip using exoglycosidases, revealing fractions containing structures capable of binding a cancer-specific monoclonal antibody that was generated in-house, mAb A4. Electrospray ionization mass spectrometry was then used to structurally characterize target glycans in bound fractions, thereby identifying actual, full-sized cancer-associated glycan structures responsible for mAb A4 binding. To the best of our knowledge, this study is the first to fabricate shotgun glycan arrays directly from cancer cell line glycans for the identification of cancer-associated carbohydrate antigens.

## Results

### mAb A4 distinguishes cancerous from non-cancerous ovarian epithelial cell lines

**Figure 1:**
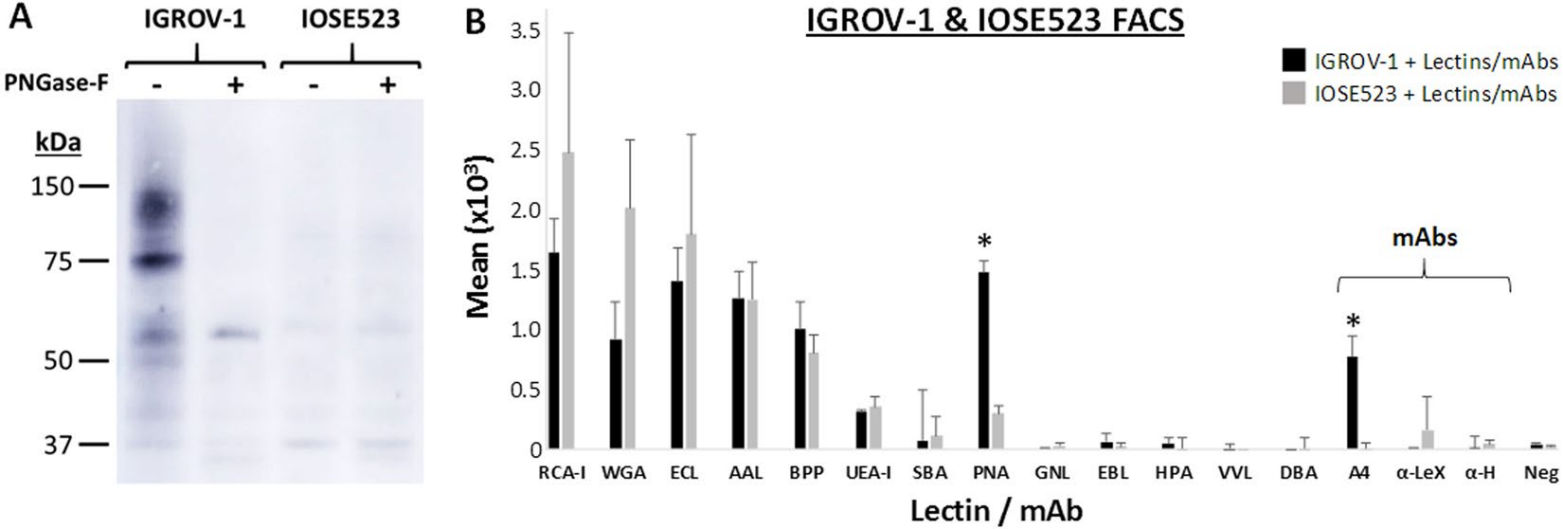
mAb A4 distinguishes cancerous from non-cancerous ovarian epithelial cell lines. (**A**) Western blotting of IGROV-1 and IOSE523 whole cell lysate shows mAb A4 binding to a multiplicity of targets in IGROV-1, but not in IOSE523 cells. When cell lysates are treated with PNGase-F, binding to IGROV-1 is mostly abolished, indicating that mAb A4 recognizes primarily N-linked glycans. A full-length blot is included as Supplementary Fig. 10. (**B**) FACS experiments with a panel of plant-derived lectins and sugar-specific antibodies shows that only mAb A4 and peanut agglutinin are able to positively distinguish between cancerous and non-cancerous ovarian epithelial cell lines (N = 3), suggesting that mAb A4 may bind to ovarian cancer-associated glycans. Abbreviations: RCA-I = ricinus communis agglutinin-1, WGA = wheat germ agglutinin, ECL = erythrina cristalli lectin, AAL = aleuria aurantia lectin, BPP = bauhinia purpurea lectin, UEA-I = Ulex Europaeus-1; SBA = soybean agglutinin, PNA = peanut agglutinin, GNL = galanthus nivalis lectin, EBL = elderberry bark lectin/sambucus nigra agglutinin; HPA = helix pomatia agglutinin, VVL = vicia villosa lectin, DBA = dolichol biflorous agglutinin, A4 = mAb A4, α-Lex = anti-Lewis X antibody, α-H = anti-H type I antibody, Neg = negative control. *Represents p < 0.05, Bonferroni corrected Student’s T-test.

Western Blot experiments were first carried out to determine if mAb A4 bound a specific class of glycans in IGROV-1 ovarian carcinoma cell lines. Figure 1A shows that mAb A4 bound to IGROV-1 cell lysate in a “smear” pattern characteristic of many carbohydrate-specific antibodies, but not to the lysate of a control ovarian epithelial cell line, IOSE523. Antibody binding was largely abolished by treating IGROV-1 cell lysate with PNGase-F, suggesting that mAb A4 mainly targeted N-glycans. To confirm that mAb A4’s ability to distinguish between the two cell types was not a trivial side-effect of lower overall expression of N-glycans in one cell type relative to the other, we performed live cell fluorescence activated cell sorting (FACS) experiments using a panel of plant-based lectins and carbohydrate-specific antibodies (Fig. 1B). Our results showed that only mAb A4 and peanut agglutinin could distinguish between the two cell types. Peanut agglutinin has been reported as being capable of binding specifically to ovarian carcinoma cells^32^, but recognizes primarily O-glycans (T and Tn antigen) instead of N-glycans. Since all other reagents bound to cell types to similar extents, this showed that the ability of mAb A4 to distinguish between cancerous and non-cancerous ovarian epithelial cells was not trivial, and could not be attributable to large-scale differences in N-glycan expression.

### mAb A4 binds to glycans containing type I N-acetyl-lactosamine

**Figure 2:**
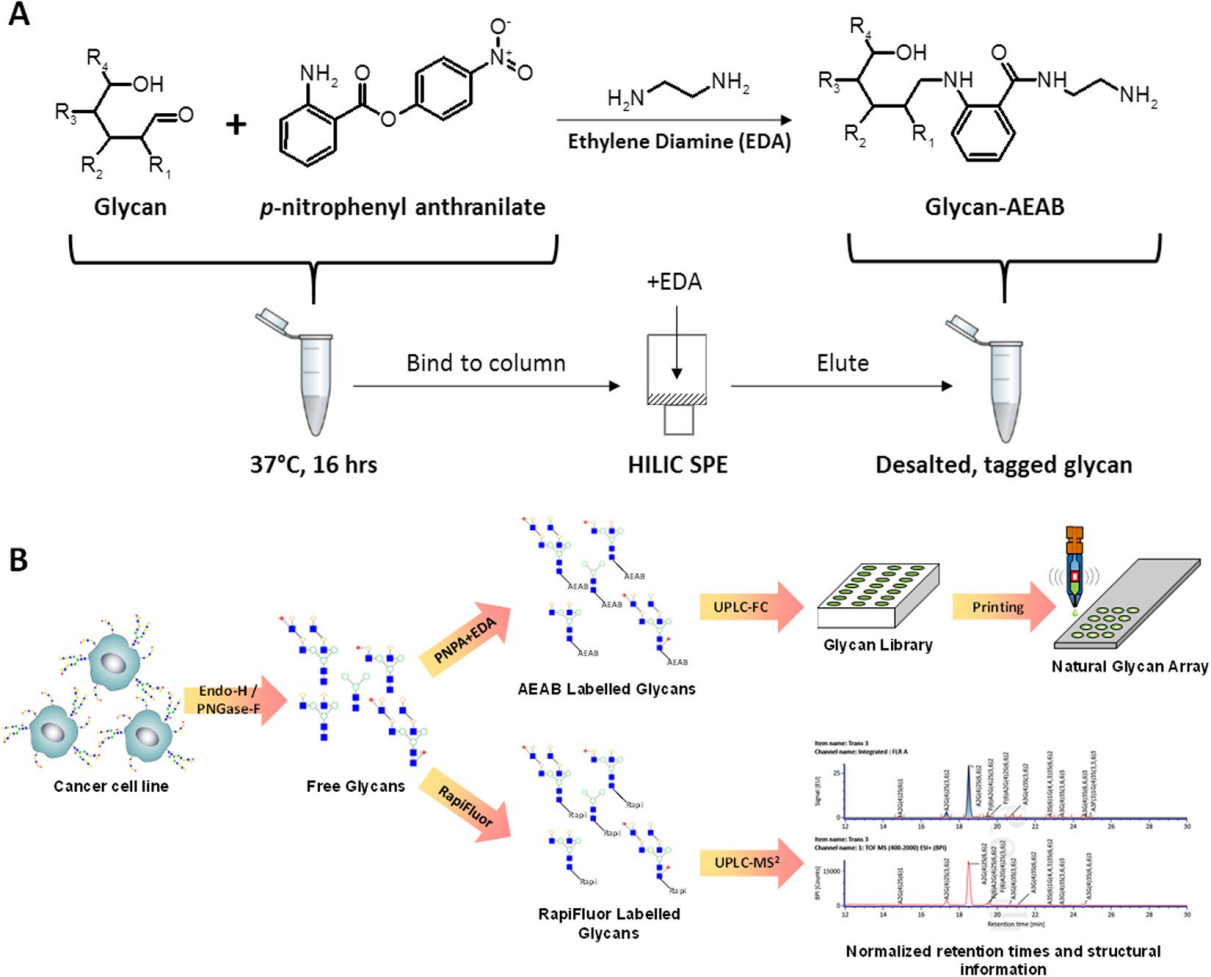
Schema of glycan tagging and printing of cancer cell line shotgun glycan microarray. (**A**) One-pot tagging of reducing glycans with bifunctional fluorescent linker 2-amino-N-(2-amino-ethyl)-benzamide (AEAB). The precursor p-nitrophenyl anthranilate is covalently bonded to reducing glycans under standard reductive amination conditions. The resulting molecule is bound to a hydrophilic cartridge (HILIC SPE), then reacted on-column with ethylene diamine. After washing off excess reagents, desalted glycan-AEAB is eluted from the column for analysis and microarray printing. (**B**) Complex N-glycans are extracted from cancer cell line lysate by first removing high mannose and some hybrid type N-glycans with Endo-H, then treating the remaining glycopeptides with PNGase-F. Complex N-glycans are tagged with AEAB as illustrated in part (A), or with RapiFluor. AEAB labelled glycans are fractionated using a UPLC tandem fraction collector to yield a glycan library, which is then printed as a shotgun glycan microarray. RapiFluor labelled glycans are analyzed by UPLC-MS^2^ to yield structural information at each normalized retention time. Normalized retention times are then used to match structural information to shotgun glycan microarray fractions.

Next, chemically defined microarrays was prepared by tagging off-the-shelf glycan standards with bi-functional dye N-(aminoethyl)-2-amino benzamide (AEAB)^20,33^ (Fig. 2A; see Supplementary Table 1 for a full list of structures). AEAB-tagged glycans were assessed for purity and concentration using HILIC-UPLC (Supplementary Fig. 1), and the resulting chemically defined microarrays were validated using a set of lectins and glycan-specific antibodies with well-defined specificities (Supplementary Fig. 2). See Supplementary Fig. 3 for a brief guide to the Oxford notation for representing glycan structures.

**Figure 3:**
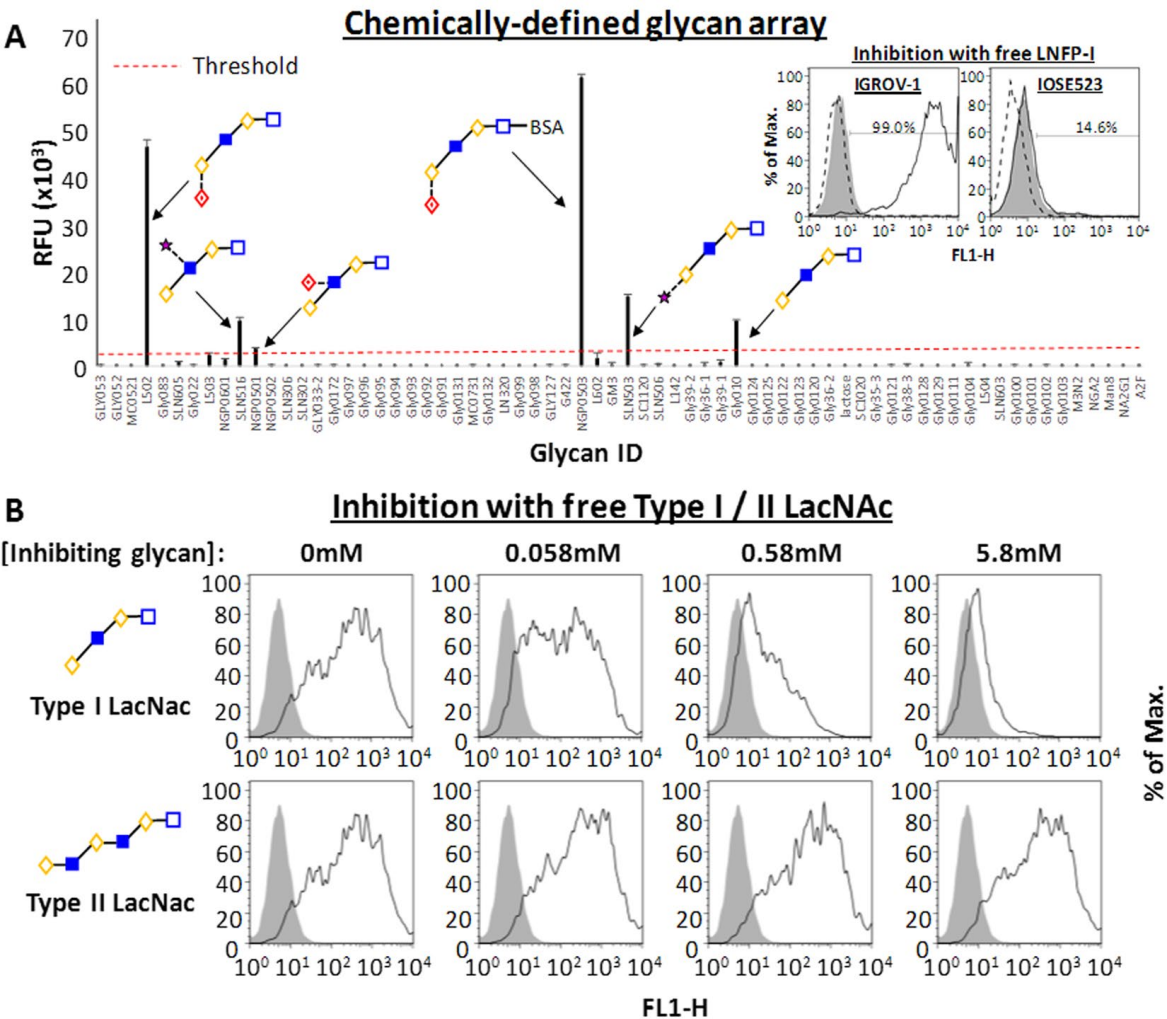
mAb A4 analysis using chemically-defined glycan array, and verification of hits with FACS. (**A**) Chemically-defined glycan array shows that mAb A4 binds to structures containing Gal-(β1-3)-GlcNAc (type I LacNac), and that terminal α1-2 fucose (H-antigen) appears to greatly increase binding intensity. H-type I antigen is therefore identified as the primary binding target, and type I LacNAc as the secondary binding target. Inset: 5.86 mM free lacto-N-fucopentose I (LNFP-I) blocking oligosaccharide abolished mAb A4 binding to IGROV-1 cells, verifying that H-type I is a ligand for mAb A4. IOSE523 cells showed only background binding (14.6% positive) of mAb A4 that could not be abolished by free LNFP-I. Solid black line represents cells + mAb A4; shaded area represents cells + mAb A4 + 5.86 mM LNFP-I blocking oligosaccharide; dashed black line represents cells without antibody. (**B**) Free glycans containing type I LacNAc, but not type II LacNac (Gal-(β1-4)-GlcNac), inhibit binding of mAb A4 to IGROV-1 cells in a dose-dependent fashion. Solid back line represents cells + mAb A4 + free inhibiting oligosaccharide; shaded area represents cells without antibody.

Microarray hybridization experiments showed that mAb A4 bound with varying intensities to at least 6 structures (Fig. 3A) with a coefficient of variation ranging from 0.99–7.82% (N = 3). An analysis of the bound structures revealed the common motif Gal-β1,3-GlcNAc-β1,3-Gal-β1,3-Glc, a tetrasaccharide containing type I N-acetyl-lactosamine (Gal-β1,3-GlcNAc; LacNAc). Moreover, the presence of terminal α1,2 fucose appeared to substantially increase the binding intensity, suggesting that mAb A4’s primary target is H-type I antigen (Fuc-α1,2-Gal-β1,3-GlcNAc), with secondary binding to type I LacNAc. We verified that H-type I antigen was a biologically relevant ligand of mAb A4 by showing that free lacto-N-fucopentose I (Fuc-α1,2-Gal-β1,3-GlcNAc-β1,3-Gal-β1,3-Glc) inhibited the binding of mAb A4 to IGROV-1 cells (Fig. 3A, inset). We then sought to verify that mAb A4 specifically recognized type I LacNAc. Free oligosaccharides containing either type I LacNAc or type II LacNAc (Gal-β1,4-GlcNAc) as potential inhibitors of mAb A4 were applied over a range of concentrations (Fig. 3B). Our experiment confirmed that only type I, but not type II LacNAc, could inhibit mAb A4 binding to IGROV-1 cells in a dose-dependent manner.

### Extraction and fractionation of IGROV-1 N-glycans for shotgun glycan microarray fabrication

**Figure 4:**
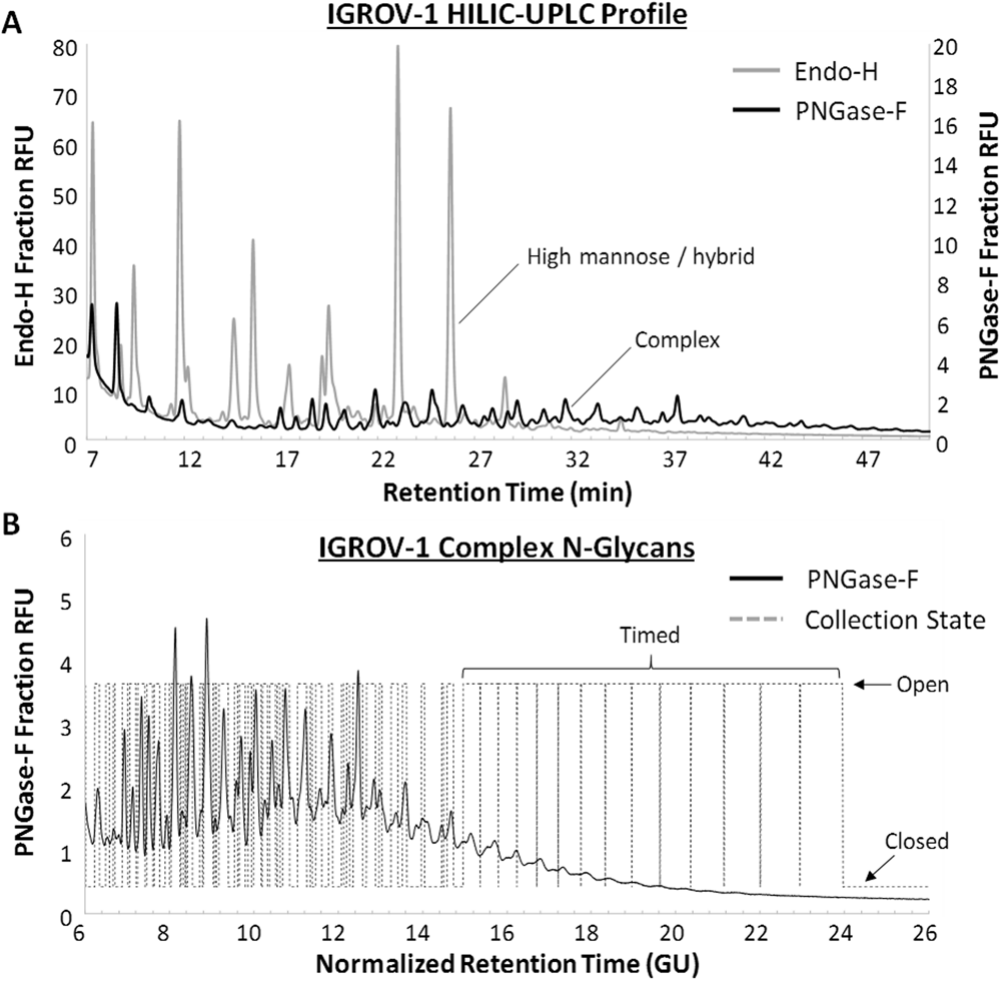
Fractionation of complex N-glycans from IGROV-1 cell lysate. (**A**) IGROV-1 N-glycome is dominated by high mannose/hybrid type structures. High mannose/hybrid structures were first released from tryptic glycopeptides using Endo-H (grey trace; left ordinate axis), and then PNGase-F was used to release complex N-glycans from the remaining glycopeptides (black trace; right ordinate axis). HILIC-UPLC chromatograms showing the relative intensities of both profiles are overlaid. (**B**) Complex N-glycans were separated into 59 fractions using a fraction collector. Slope and threshold based collection was possible until fraction 45, after which time-based collection at 1-minute intervals was used due to low glycan abundance. Solid black trace: complex N-glycans; Dashed black trace: fraction collector collection state (open or closed).

To determine the full structures of cancer-associated glycan targets and ascertain their relative contributions to mAb A4 binding, we extracted, tagged and fractionated IGROV-1 N-glycans according to the schema shown in Fig. 2B. Consistent with published literature^34,35^, the N-glycome of IGROV-1 cells was found to be dominated by high mannose glycans which, if not removed would significantly contaminate many of the collected fractions. Accordingly, IGROV-1 cell lysate was pre-treated with Endo-H, specifically releasing high mannose and some hybrid N-glycans, before subjecting the remaining glycopeptides to PNGase-F treatment to release complex N-glycans. Fluorescently labelled high mannose/hybrid N-glycans yielded a much higher maximum peak height of 78 EU as compared to 4.5 EU for complex N-glycans (Fig. 4A), showing that they were present in far greater quantities. Complex N-glycans were then fractionated using either slope and threshold detection (fractions 1–45) or time-based collection (fractions 46–59) using a Waters Fraction Collector III optimized for small sample collection (Fig. 4B; see Supplementary Fig. 4 for fraction collector optimization). Slope and threshold detection was found to be superior to time-based collection because it was robust to chromatographic drift and enabled the collection of individual peaks; however, time-based collection was necessary to collect the very low abundance glycans eluting at a normalized retention time ≥ 15 Glucose Units (GU). Although many of these peaks were near to or even below the limit of detection, they were nevertheless later found to contribute greatly to mAb A4 binding.

### Hybridization of mAb A4 to shotgun glycan microarray

**Figure 5:**
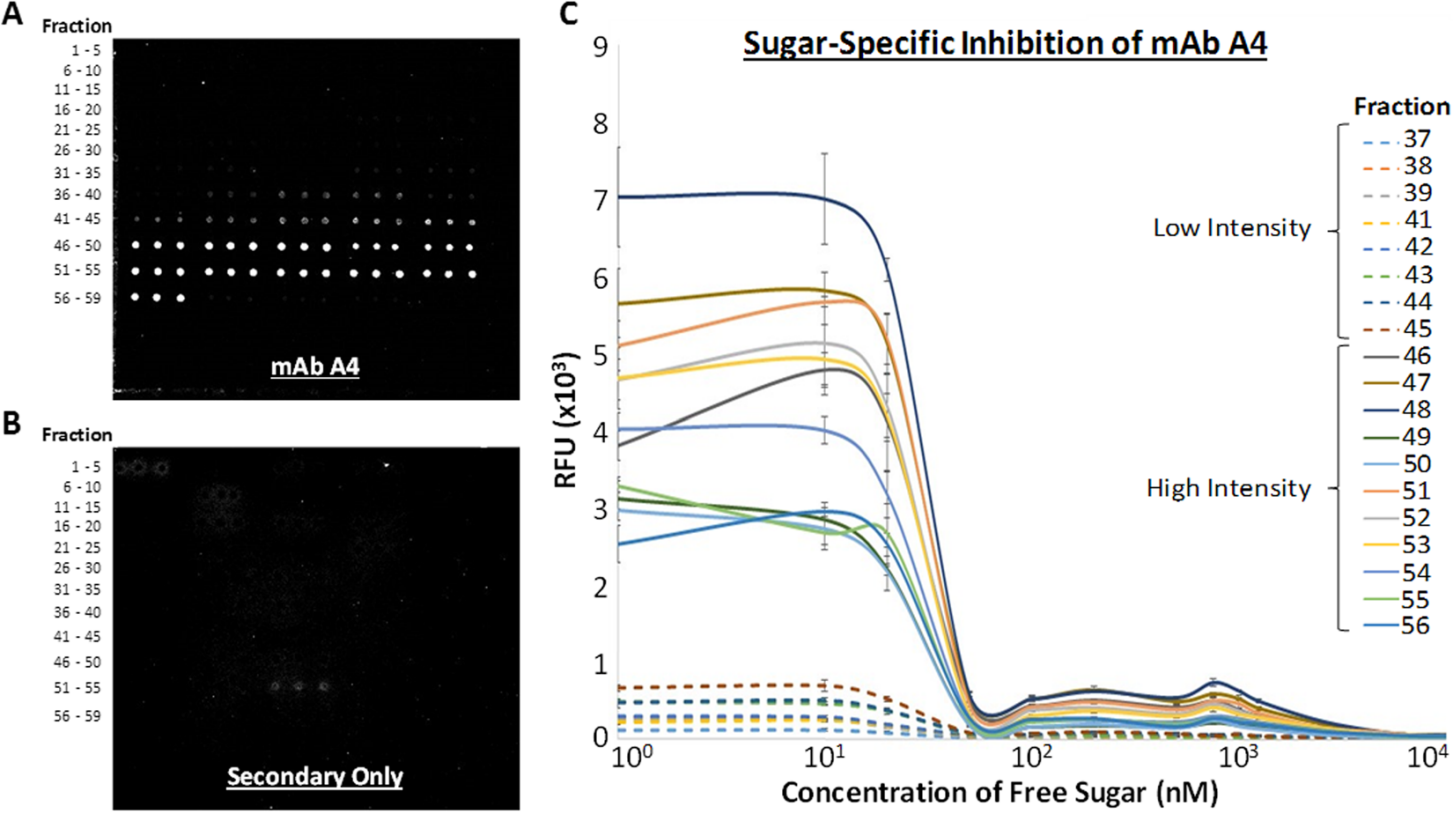
Carbohydrate-specific binding of mAb A4 to IGROV-1 complex N-glycan array. (**A**) mAb A4 bound weakly to fractions 37–39 and 41–45, and strongly to fractions 46–56. (**B**) Limited background binding to fraction 53 was seen in the negative control (secondary antibody only). (**C**) mAb A4 binding was shown to be sugar-specific as free LNFP-I glycan inhibited binding in a dose-dependent fashion. All glycan fractions were printed in triplicate.

mAb A4 bound with high signal intensity and low background noise to the later fractions of the IGROV-1 shotgun glycan microarray (Fig. 5A, B). The bound spots could be divided into 2 categories: low intensity fractions (fractions 37–39, 41–45), and high intensity fractions (fractions 46–56). As mentioned in the previous paragraph, this was remarkable because the glycans eluting in fractions 46–56 were present in very small quantities compared to the rest of the complex N-glycans, yet they were observed to contribute most of the binding intensity on the microarray. The coefficients of variation for high intensity fractions ranged from 0.75–15.07% (N = 3), whereas the coefficients of variation for low intensity fractions was higher, ranging from 10.87–36.00% (N = 3).

Since H-type I antigen was previously identified as a strong putative ligand, and other type I LacNAc containing structures as weak ligands, we hypothesized that differences in binding intensity could be due to the presence of H-type I antigen in high intensity fractions and type I LacNAc in low intensity fractions. If this were the case, we hypothesized that mAb A4 would bind with lower affinity to low intensity fractions. After determining that 20 µg/ml antibody was on the upper end of the binding curves for both low and high-intensity fractions (Supplementary Fig. 5), a binding inhibition assay using free lacto-N-fucopentose I oligosaccharide was performed at this antibody concentration to determine the IC_50_ for each fraction (Fig. 5C). To our surprise, both high intensity and low intensity binders exhibited similar dose-dependent inhibition curves with IC_50_ of approximately 30 nM, suggesting that mAb A4 bound both low and high intensity fractions with similar affinity. This result suggested that the same ligands were responsible for binding in all fractions, but were present in different abundances.

### On-slide exoglycosidase sequencing of IGROV-1 glycan targets

**Figure 6:**
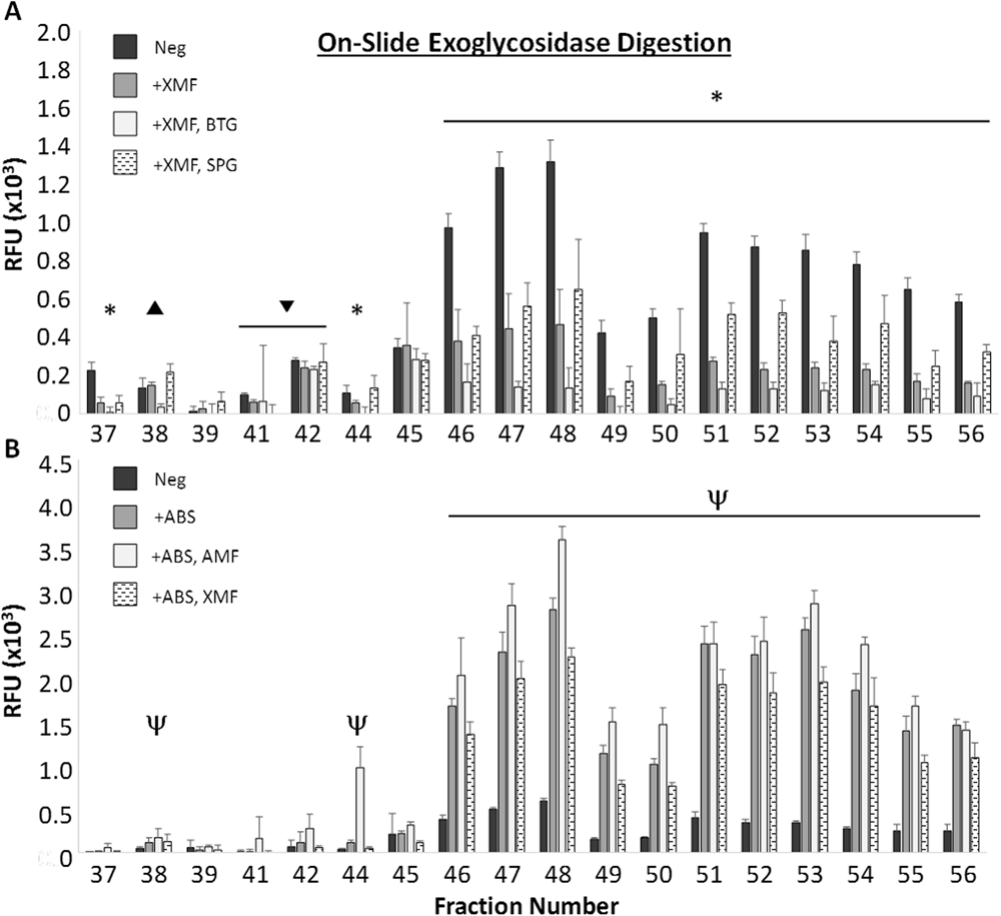
On-slide exoglycosidase digestions reveal the contributions of H-type I and sialyl-LacNac to mAb A4 binding. (**A**) Most fractions (37, 44, 46–56) showed a decrease in mAb A4 binding after on-slide digestion with XMF. There was a further decrease in binding intensity with subsequent BTG digestion which was not seen with SPG digestion, indicating that Fuc-(α1-2)-Gal-(β1-3)-GlcNAc i.e. H-type I was the main contributor to binding. In fractions 41 and 42, a statistically significant binding decrease was only seen with XMF treatment but not with BTG or SPG, allowing us to conclude only that terminal α1-2 fucose is involved in binding to these fractions. Finally, fraction 38 showed a statistically significant binding decrease only with BTG treatment, but not with XMF or SPG, indicating that type I LacNAc is primarily responsible for binding to this fraction. * = decrease in signal with XMF and BTG, ▼ = decrease in signal with XMF only, ▼ = decrease in signal with BTG only, p < 0.05 Student’s T-test. (**B**) Fractions 38, 44, 46–56 showed large increases in binding intensity after on-slide digestion with ABS sialidase, which was only partially abolished by subsequent XMF, but not AMF, digestion. This suggests that the presence of sialic acid restricts binding to poly-LacNac within these glycan structures, and that H-antigen is only a minor binding determinant in de-sialylated structures. ψ = increase in signal with ABS treatment, p < 0.05 Student’s T-test. XMF = xanthomonas manihotis fucosidase (α1,2 fucosidase); BTG = bovine testis galactosidase (β1-3,4 galactosidase); SPG = streptococcus pneumoniae galactosidase (β1-4 galactosidase); ABS = arthrobacter ureafaciens sialidase (α2-3,6,8,9 sialidase); AMF = almond meal fucosidase (α1-3,4 fucosidase); Neg = negative control.

To conclusively determine if H-type I antigen was indeed the primary mAb A4 ligand, conditions for on-slide exoglycosidase sequencing was optimized (Supplementary Figs 6, 7) and applied to the shotgun glycan microarray. Treatment with Xanthomonas Manihotis Fucosidase (XMF; Fig. 6A), an α1–2 fucosidase, significantly reduced mAb A4 binding to all high-intensity binding fractions, as well as to fractions 37 and 44. Treatment with XMF in combination with Bovine Testis Galactosidase (BTG), a β1-3,4 galactosidase, further reduced binding intensity; however, treatment with XMF in combination with Streptococcus Pneumonia β-Galactosidase (SPG), a β1-4 galactosidase, did not. Taken together, this experiment clearly identifies Fuc-α1,2-Gal-β1,3- as the terminal glycan motif primarily responsible for mAb A4 binding, showing that H-type I antigen was the primary mAb A4 ligand in most fractions. Further experiments showed that digestion with Arthrobacter Ureafaciens Sialidase (ABS), an α2-3,6,8 sialidase, greatly increased binding to all high-intensity binding fractions (Fig. 6B). This increase in signal could only be partially abolished by treatment with XMF, but not with Almond Meal Fucosidase (AMF), an α1-3,4 fucosidase, suggesting that increased accessibility of mAb A4 to terminal fucosylated structures such as Lewis A or Lewis X could not account for the increase in binding intensity. Instead, this experiment suggested that high intensity binding fractions possessed type I (poly)LacNAc structures which were capped with sialic acid, and which were therefore relatively inaccessible to mAb A4 binding. Desialylation upon treatment with ABS increased the accessibility to type I (poly)LacNAc, but without this intervention type I LacNAc is only a minor contributor to antibody binding.

### ESI-MS^2^ structural characterization of mAb A4 binding targets

**Figure 7:**
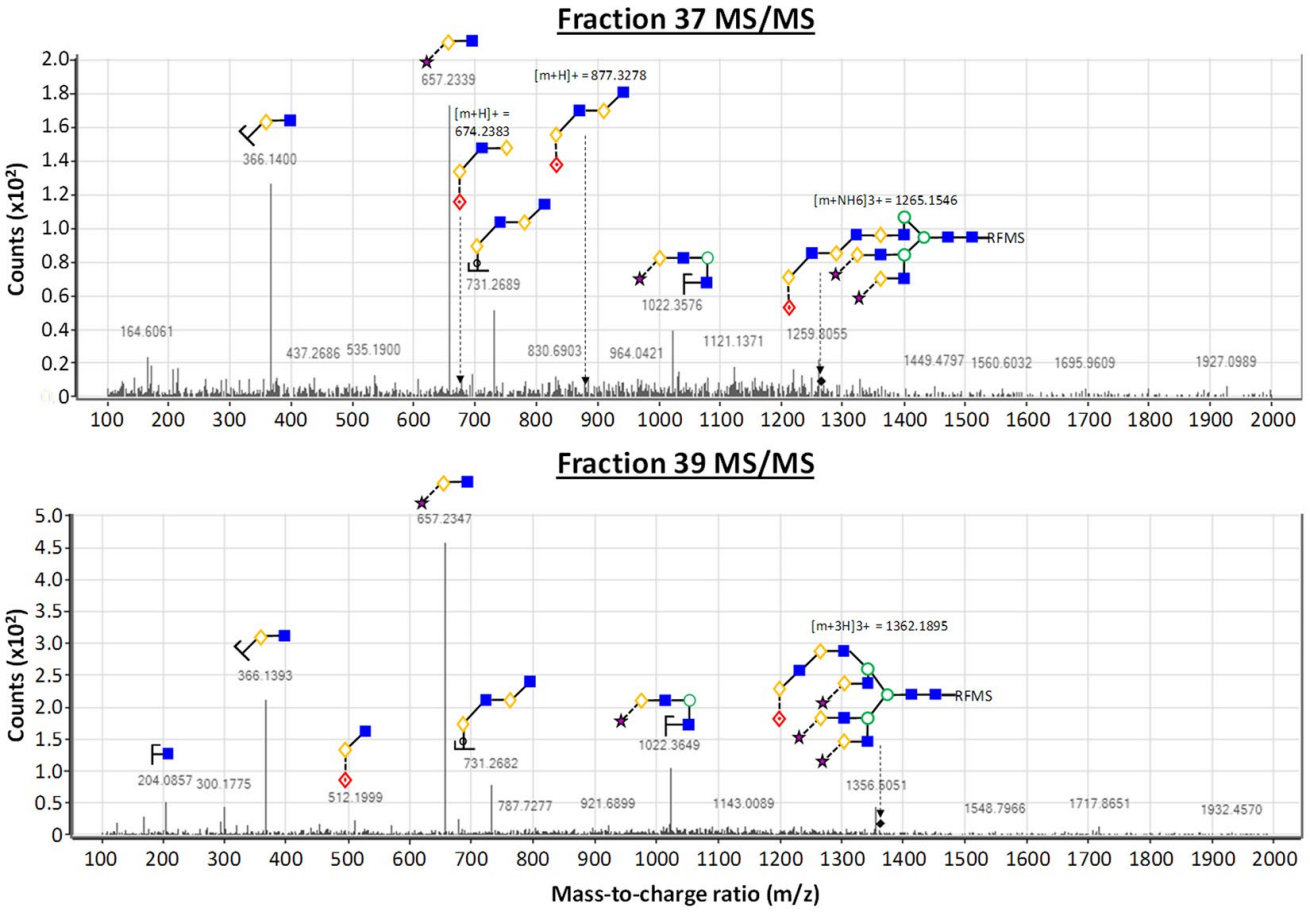
MS/MS structural characterization of putative glycan biomarkers. Positive mode fragmentation of putative glycan biomarkers in fractions 37 (top panel) and 39 (bottom panel) revealed LacNAc and fucosylated LacNAc fragments that are consistent with chemically-defined and natural glycan microarray experiments.

To elucidate the full structures of mAb A4 ligands, ESI-MS^2^ was performed. In our hands, we found that the AEAB tag greatly suppressed ionization efficiency (data not shown). Given the low abundance of many of the glycans of interest, fresh samples were tagged with Waters *Rapi*Fluor-MS, a proprietary dye that has been reported to increase the ionization efficiency of glycans by 160-fold relative to conventional tagging with 2-aminobenzamide^36^. After optimizing the amount of input glycoprotein for the labelling experiment (Supplementary Fig. 8), we obtained useful ESI-MS^2^ data in fractions 37 and 39 (Fig. 7). Later fractions contained progressively lower abundances of glycans that could not characterized satisfactorily (Supplementary Fig. 9). Fractions 37 and 38 both contained sialylated, tri- or tetra-antennary structures with poly-LacNAc arms terminating in H-type I antigen, which is fully consistent with our shotgun glycan microarray, chemically defined glycan array and live cell binding experimental data.

## Discussion

There is a pressing need for antibodies which may be useful for therapy or early detection of ovarian cancer. In 2016, the American Cancer Society estimates that there will be 22,280 new cases of ovarian cancer and 14,240 deaths resulting from this disease in the United States alone, making it the fifth most common cause of cancer-related death of American women^37^. Worldwide, 239,000 new cases were diagnosed in 2012. Although ovarian cancer is highly treatable if caught at an early stage, approximately 75% of epithelial ovarian carcinomas present with locally advanced or disseminated disease at the time of diagnosis, making it the most lethal gynecological malignancy^38^. Currently available cancer-targeting antibody therapeutics are typically raised in a highly-directed manner against protein targets known to be (over)expressed in cancers, such as HER2^39^ or EGFR^40^. In contrast, mAb A4 was raised using an undirected approach by immunizing against live human embryonic stem cells^41^, instead of against a known target. This approach does not require the investigator to have a deep knowledge of the surface antigen expression on cancer cells because it takes advantage of the host’s immune system to generate antibodies against a multitude of targets. Hybridoma libraries can then be created to test the cancer-targeting capabilities of each antibody. On the other hand, further development of promising antibodies may be hindered by the obscurity of the target ligand, as in the case of mAb A4, requiring sophisticated analyses to elucidate.

Over the past 30 years, experiments involving immunization with live cells or cell extracts^13–15,42–45^ have also produced antibodies with the capacity to specifically target cancer or stem cells. Gildersleeve *et al*.^45^ have shown that glycan-binding antibodies are commonly produced in such experiments, with 41% of hybridoma antibodies targeting carbohydrates. This may be somewhat surprising given the pharmaceutical industry’s experience with carbohydrate vaccines has shown that carbohydrates elicit relatively poor immune responses, especially in the young and the elderly, and require techniques such as conjugation to an immunogenic carrier protein for satisfactory responses^46–48^. Nevertheless, almost all cell surface or secreted proteins are glycosylated, and different classes of glycans may bear similar structures, such as the ABO or Lewis antigens^49^. Consequently, cancer-associated glycan structures may represent a much higher molar abundance on the cell surface than cancer-associated proteins, thus explaining their higher than expected immunogenicity.

In this study, shotgun glycomics^20,21^ approach was applied to identify the glycans targeted by mAb A4. Published reports utilizing the shotgun glycomics or “designer” microarray approach have typically sourced glycan probes from carbohydrate-rich sources such as human milk^23^, pig lung^21^ or intestinal mucins^31^. In contrast, for this study source glycan probes were sourced directly from cancer cell lines because the targets we sought were likely to be present in exceedingly small quantities in cancer cell lines^31^, which might complicate antibody binding studies or mass spectrometric characterization. However, in our view the many benefits of sourcing glycan probes directly from cancer cell lines has outweighed the drawbacks: we were able to uncover cell-specific features such as the outsized contribution of late-eluting fractions (fractions 46–56) to mAb A4 binding despite their comparatively low abundance; we were able to show the existence of high and low-binding fractions and propose a mechanism for this phenomenon; and were able to examine the relative contributions of type I LacNac and H-type I antigen to binding, both of which were found to be valid targets of mAb A4. Most importantly, our findings are more relatable to cancer biology because we chose to use cancer cell-derived glycan probes.

To overcome the difficulties caused by working with such small glycan quantities, we made some modifications to the shotgun glycomics protocol pioneered by Cummings *et al*.^20,21^: firstly, one-dimensional HILIC-UPLC separation was used to fractionate the glycan probes instead of the two-step approach of normal phase HPLC followed by porous graphitic carbon favored by Cummings *et al*. This allowed us to reduce the number of sample handling steps and avoid the potential sample loss through incomplete elution of highly sialylated glycans from the strongly retentive porous graphitic carbon column^30^. Secondly, we relied heavily on binding inhibition assays and on-slide exoglycosidase sequencing to positively identify the ligands of interest since these assays relied on fluorescent detection and were therefore generally more sensitive than mass spectrometry^50–52^. Thirdly, we used Waters RapiFluor-MS^53^ to increase ionization efficiency and facilitate structural characterization of the relevant glycan targets. These innovations made it possible for us to derive useful data from our experiments despite the low abundance of glycan targets.

This study’s main conclusion is that the expression of H-type I antigen on ovarian carcinoma cell line IGROV-1 mediates the ability of mAb A4 to distinguish between cancerous and non-cancerous ovarian epithelial cells. Separately, although type I LacNAc was found to be present in several mAb A4-binding fractions, it was much less available for binding than H-type I antigen due to capping with sialic acid. When treated with sialidase, the observed mAb A4 binding signal was far more intense, and in fact binding to type I LacNAc was found to dominate over binding to H-type I antigen. This is consistent with studies by Bertozzi *et al*.^54^ and others^55^ which show that increased sialylation decreases the susceptibility of cells to immune clearance and contributes to cancer cell immune evasion. It is interesting to note that the presence of terminal α1,2 fucose in H-type I antigen makes it impossible for the cell to “cap” this antigen with sialic acid, which may have made it easier for the immune system to target.

H-antigen expression is primarily reliant on FUT-1^56^, which is necessary to form terminal α1,2 fucose in non-secretors. β3GalT-1, 2 and 5 are the three main glycosyltransferases responsible for synthesizing type 1 LacNAc on N-glycans^57–59^ Although there has been some controversy about the ability of β3GalT-5 to act on N-glycan substrates^59^, other studies have shown that it is capable of doing so^57^; moreover, it appears to have the strongest enzymatic activity of the three enzymes^58^. Recently, Choo *et al*.^19^ used siRNA knockdown experiments that inhibition of β3GalT-5 is sufficient to abolish mAb A4 binding to ovarian carcinoma cells because it reduces the expression of H-type 1 and type 1 LacNAc, further supporting the findings of this study. Multiple studies have shown that the over-expression of H-antigen and Lewis Y are associated with advanced pathological stages, and are involved in cell proliferation, migration and invasion. Lin B *et al*.^60^ showed that c-Jun, a transcription factor linked to cancer malignancy, transcriptionally regulates expression of FUT-1 in ovarian cancer. Nozawa *et al*.^61^ showed that over-expression of FUT-1 in ovarian carcinoma-derived cells increased their adhesion to mesothelial cells and increased cell viability in the presence of anti-cancer drug 5-fluorouracil. β3GalT-5 has been shown to be the enzyme primarily responsible for the synthesis of CA19.9 (sialyl-Lewis A), an established biomarker of pancreatic and colon cancer^62^. Moreover, recent studies have also suggested that it is a biomarker for gynecological cancers^63^. Knowledge of the glycan targets of mAb A4 will therefore facilitate its clinical translation and provide another tool to study ovarian cancer oncogenesis and glycobiology.

## Experimental Procedures

### Materials

Unless stated otherwise, all chemicals were purchased from Sigma Aldrich. Glycan standards were purchased from Dextra Laboratories and Elicityl. A full list of these glycan standards and their structures is shown in Supplementary Table 1. *P*-nitrophenyl anthranilate was purchased from Matrix Scientific. HILIC SPE columns (Glykoclean S cartridges) were purchased from Prozyme. Porous graphitic carbon SPE columns (Hypercarb) were purchased from Thermo Fisher Scientific. C18 Sep-Pak SPE columns were purchased from Waters. PD Minitrap G-10 size exclusion columns (MWCO 700 Da) were purchased from GE Healthcare. PNGase-F (P0705L, glycerol-free), Endo-H (P0702L), 10x denature buffer, 10% NP40, and 10x Glycobuffers 1,2 and 3 were purchased from New England Biolabs. Exoglycosidases AMF and XMF were purchased from New England Biolabs (P0724L, P0769L); ABS, BTG and SPG were purchased from Prozyme (GK80040, GKX-5013, GKX-5014). NHS-coated slides (Nexterion Slide H) were purchased by special order from SCHOTT Technical Glass Solutions. Plant-derived biotinylated lectins were purchased from Vector Laboratories. Anti-CD15 (13-0159-82) and Anti-H-type-I (13-9810-82) biotinylated monoclonal antibodies were purchased from eBiosciences. mAb A4 was produced and provided by Dr. Andre Choo as outlined below.

### Generation of mAb A4

Cancer-targeting antibody hybridomas were generated from mice immunized with live human embryonic stem cells^41^. Hybridomas were maintained in ClonaCell Medium E (Stemcell, Vancouver, BC, Canada). mAb A4 was selected for further studies after high throughput FACS analyses showed that it bound to many cancer cell lines, including breast, ovary and kidney, whereas it did not bind to normal cell lines. mAb A4 IgM was purified in-house by steric exclusion chromatography^64^.

### High-throughput FACS

IGROV-1 cells (p30–40) were cultured in RPMI 1640 supplemented with 10% FBS; IOSE523 cells (p17+) were cultured in MCDB105 and M199 1:1 supplemented with 10% FBS. Cells were defrosted from liquid nitrogen frozen stocks and passaged twice in T-175 flasks prior to release with 0.05% Trypsin-EDTA (Thermo Fisher Scientific 25300054) for FACS analysis. 0.15 × 10^6^ cells were resuspended in FACS buffer (1x phosphate buffered solution, pH 7.4, supplemented with 1% bovine serum albumin) containing 20 µg/ml lectin or monoclonal antibody for 1 h at 4 °C. Cells were washed twice in FACS buffer with centrifugation at 400xg for 5 min, then incubated with 10 µg/ml Streptavidin-488 (Thermo Fisher Scientific, S11223) for 45 min. Cells were then washed thrice in FACS buffer and analyzed on a BD FACSCalibur flow cytometer with high throughput sampler. Data was analyzed with FlowJo software (version 7.4), and are shown as representative histograms or as mean fluorescence values ± standard deviation (N ≥ 3 biological replicates).

### Cell Lysis and Glycoprotein Extraction

15 × 10^6^ IGROV-1 or IOSE523 cells were suspended in 600 µl 50 mM ammonium bicarbonate, pH 8.5, containing 1% CHAPS, placed in an ice bath and lysed using a Misonix 3000 ultrasonic cell disruptor (3 × 15 sec, power setting 2). Insoluble debris was pelleted by centrifugation at 1400xg for 30 min at 4 °C. The clarified cell lysate was then either frozen as aliquots for future use, or directly used for Western Blot (see below) or N-glycan release.

### Western Blot

Cell lysate protein concentration was determined using BCA assay (Thermo Fisher Scientific 23225). 50 µg of cell lysate protein was denatured by adding 10x glycoprotein denature buffer and heating at 70 °C for 15 minutes. Denatured cell lysate proteins were mixed with 4x LDS sample buffer (NuPAGE), then 30 µg of protein was loaded into each lane of Novex 4–12% Bis-Tris gels and run at 120 V for 2 h at 4 °C. Glycoproteins were transferred from their gels to PVDF membranes using the iBlot dry blotting system (ThermoFisher Scientific) following the manufacturer’s recommended settings. Transferred blots were first washed in MilliQ water, then blocked for 1 hour with a 5% BSA solution in Tris-buffered saline + 0.1% Tween-20. mAb A4 was applied at 0.1 µg/ml overnight at 4 °C, and HRP-conjugated goat anti-mouse IgM (ThermoFisher Scientific) at a dilution of 1:20,000 at room temperature for 1 hour. Blots were washed extensively in Tris-buffered saline + 0.1% Tween-20 between each antibody application step. Bands were visualized with SuperSignal West Pico Chemiluminescent substrate and imaged using an ImageQuant LAS 500 (GE Healthcare Life Sciences).

### Enzymatic N-glycan Release

For Western Blots, N-glycan release was effected after heat denaturation (see above). 10% NP40 was added to denatured cell lysates in a 2:1 v/v ratio relative to the volume of 10x denature buffer used, followed by 10x Glycobuffer 2 and MilliQ water to bring the final denatured protein concentration to 1 µg/µl. PNGase-F was then added at a concentration of 50U per microgram of denatured cell lysate protein and allowed to react overnight at 37 °C.

For shotgun glycan microarrays, 20 µg of mass spectrometry grade trypsin (Promega V5280) was added to 600 µl of clarified cell lysate and allowed to incubate overnight at 37 °C. Dithiothreitol was then added to a final concentration of 40 mM, and the tryptic digest was then heat-denatured at 95 °C for 15 min. After correction of sample pH to 6.3 using 5% acetic acid solution, one-tenth the total volume of 10x Glycobuffer 3 was added to the solution followed by 1000U of Endo-H, and reaction was allowed to proceed overnight at 37 °C. Liberated high mannose/hybrid N-glycans were separated from remaining (glyco)peptides using C18 Sep-Pak columns as reported elsewhere^65^. For PNPA labelling, high mannose/hybrid N-glycans were further desalted using Hypercarb columns^66^. After elution from C18 Sep-Pak columns, the remaining (glyco)peptides were dried in a centrifugal evaporator and reconstituted in 250 µl 1x Glycobuffer 2. 1500U PNGase-F was then added and allowed to react overnight at 37 °C. Liberated complex N-glycans were separated from remaining peptides using C18 Sep-Pak columns, and further desalted using Hypercarb columns.

### PNPA Labelling of Released N-glycans and Glycan Standards

PNPA labelling was carried out in a one-pot reaction, largely as described by Song *et al*.^33^ but with some modifications. PNPA labelling reagent consisting of 0.3 M PNPA and 1 M sodium cyanoborohydride was prepared by dissolving the respective chemicals in a mixed solvent of 70% anhydrous DMSO and 30% glacial acetic acid. 20 µg of dried glycan standard, or released N-glycans from 15 × 10^6^ cells, was dissolved in 100 µl PNPA labelling reagent and allowed to react overnight at 37 °C on a dry heating block. To introduce an ethylene diamine linker, thereby converting PNPA to AEAB, the labelled glycans were adsorbed to Glykoclean S SPE cartridges, and excess reagents were washed off with 5 × 1 ml washes of a mixed solvent composed of 96% acetonitrile and 4% water. 2 × 200 µl of 10% ethylene diamine dissolved in acetonitrile was then added to each cartridge and allowed to react for 1 minute. Following a further 5 × 1 ml washes in 96% acetonitrile, labelled glycans were eluted into 2 ml snap-cap tubes using 4 × 400 µl MilliQ water and dried in a centrifugal evaporator.

### Characterization of Labelled Glycan Standards’ Purity and Concentration

20 µg samples of AEAB labelled glycan standards were reconstituted in 50 µl MilliQ water. 10 µl of each glycan standard solution was diluted into 20 µl of HPLC-grade acetonitrile in glass sample vials (Waters). 10 µl injections from each sample vial were analyzed on a Waters Acquity UPLC with an Acquity UPLC BEH Glycan 1.7 µm 2.1 × 150mm column. Column temperature was 40 °C; flow rate was 0.4 ml/min; a linear gradient was used to ramp the mobile phase composition from 70:30 acetonitrile:100 mM ammonium formate, pH 4.5 to 50:50 over 60 min. Chromatograms were analyzed on Chromeleon 7.2 (Thermo Fisher Scientific). Purity was calculated as the percentage area under the curve of the main peak relative to total area under all peaks in the chromatogram (Supplementary Table 1). Concentration was calculated against a calibration curve of free AEAB dye solutions of known concentrations.

### UPLC Fractionation of Labelled Released N-glycans

Prior to fraction collection, a Waters Fraction Collector III was connected in tandem to the Waters Acquity UPLC and optimized for analytical fraction collection. The default 0.5 mm inner diameter tubing was replaced with 1.6 mm outer diameter × 0.1 mm inner diameter PEEK tubing, and used to pipe fluorescence detector flow cell output to the fraction collector input. AEAB-labelled lactose was used as a standard to determine tubing dwell volume and to ensure no that band broadening was taking place (Supplementary Fig. 4). For fraction collection, AEAB labelled released complex N-glycans from 15 × 10^6^ cells were reconstituted in 60 µl of a mixed solvent composed of 67% acetonitrile and 33% water. 10 µl injections were fractionated on the Waters Acquity UPLC using the column, flow rate and mobile phase gradient as described in the previous paragraph. Fractions were collected using the following settings: EUFS = 5; 13–25 min trigger slope = 0.1 V/min, threshold voltage = 0.25 V; 25–44 min trigger slope = 0.1 V/min, threshold voltage = 0.3 V; 44–58 min timed collection at 1 min intervals.

### Glycan Microarray Printing

For shotgun glycan microarrays, fractions from 10 × 10 µl injections (see previous paragraph) were pooled, and the volatile ammonium bicarbonate salts removed by freeze-drying three times. Each desalted fraction was then reconstituted in 30 µl 0.3 M sodium phosphate buffer, pH 7.4 for printing. For chemically-defined microarrays, AEAB labelled glycan standards were reconstituted to a final concentration of 12.5 µM in 0.3 M sodium phosphate buffer, pH 7.4 for printing. Please refer to Supplementary Table 1 for a full list of structures included on the chemically-defined microarrays.

Printing was carried out as previously described^67^. Reconstituted glycans were spotted onto Nexterion H slides using a sciFlexarrayer S1 (Scienion) non-contact piezoelectric microarray printer with a volume of ~1.5 nl per spot at a relative humidity of 50%. Three replicate spots were printed for each glycan. Both types of glycan microarrays were printed as 14 subarrays per slide, consisting of either 177 (shotgun) or 204 (chemically-defined) spots per subarray. The glycans were allowed to bond covalently with the glass for ≥4 h, then microarrays were stored frozen at −20 °C. Chemically-defined glycan arrays were validated by hybridization with plant-derived biotinylated lectins of known specificity, anti-CD15 and anti-H-type-I biotinylated antibodies (Supplementary Fig. 2).

### Antibody and Lectin Hybridization on Glycan Microarrays

mAb A4 aliquots were thawed as needed and stored at 4 °C to prevent freeze-thaw damage. Plant-derived biotinylated lectins, anti-CD15 and anti-H-type-I biotinylated antibodies were reconstituted according to the manufacturer’s instructions if necessary and stored at 4 °C. All antibodies and lectins were used for glycan microarray experiments without further modifications. Prior to antibody hybridization, any remaining NHS groups on printed microarray glass surfaces were inactivated by immersion in deactivation solution (25 mM ethanolamine in 100 mM boric acid, pH 8.5, with 0.01% Tween-20). After 1 h, microarrays were washed twice in 1x PBS, then blocked with 1% BSA in 1x PBS for 1 h. Microarrays were then washed twice and hybridized with 20 µg/ml biotinylated lectins or monoclonal antibodies in 1x PBS containing Ca^2+^ and Mg^2+^ ions overnight at 4 °C. After washing three times, microarrays were hybridized with 10 µg/ml Streptavidin-Cy5 (Biolegend 405209) or goat anti-mouse IgM Dylight 650 (Thermo Fisher Scientific SA5–10153) in 1x PBS containing Ca^2+^ and Mg^2+^ ions for 1 h, washed three times and immediately visualized with a Genepix 4000B microarray scanner at 10 or 20 µm resolution in the 635 nm channel using a photomultiplier tube gain of 65 and a scan power of 100.

The resulting images were analyzed using ScanArray Express software (Perkin Elmer). Fixed circle quantification was performed after manually aligning each subarray to printed glycan spots faintly visible in the green channel due to a small amount of fluorescence from the AEAB dye. The “auto-find spots” setting was disabled, and total normalization method was used. Two representative high-resolution scans of chemically defined and shotgun microarrays, as well as their respective GAL files, have been included in the Supplementary Data. Coefficients of variation were calculated from at least three independent experiments.

### Competitive inhibition of mAb A4 binding to shotgun glycan microarrays

Fourteen independent preparations of 20 µg/ml mAb A4 in 1x PBS containing Ca^2+^ and Mg^2+^ ions were spiked with free lacto-N-fucopentose to a final free sugar concentration ranging from 0–10,000 nM, and allowed to pre-incubate for 1 h at room temperature. After shotgun glycan microarray inactivation and blocking steps as stated above, the spiked mAb A4 preparations were hybridized with microarrays overnight at 4 °C. Microarrays were subsequently washed and hybridized with goat anti-mouse IgM Dylight 650, before visualization with a Genepix 4000B microarray scanner as stated above.

### On-slide Exoglycosidase Sequencing

Digestion of glycans covalently bonded to glass generally require a higher concentration of enzyme than in-solution digests^23^. The required concentration of enzymes was optimized using chemically-defined microarrays (Supplementary Fig. 6). 2400 U/ml AMF, 4000 U/ml XMF, 4 U/ml ABS, 1.2 U/ml SPG and 3 U/ml BTG were diluted alone or in combination in 1x Glycobuffer 1 supplemented with BSA. Glycan microarrays were divided into 16 chambers using Nexterion multi-well incubation chambers (SCHOTT 1262705). 20 µl of exoglycosidase mixture was added per chamber, sealed with AlumaSeal film (Excel Scientific) and incubated overnight at 37 °C. Enzyme activity was determined by assessing the binding of Sambucus Nigra Agglutinin (for α2,6 sialic acid), Aleuria Aurantia Lectin (for fucose), or with mAb A4 to microarrays before and after exoglycosidase treatment.

### RapiFluor-MS Labelling of Released N-glycans

Optimization experiments were performed with Herceptin to determine RapiFluor-MS labelling efficiency as glycoprotein mass was increased past the recommended 15 µg limit per reaction (Supplementary Fig. 8), as stipulated in the documentation included with Glycoworks RapiFluor-MS N-glycan basic kit (Waters). For each labelling reaction, a total of 150 µg of Endo-H pre-treated (glyco)peptides containing only complex N-glycans were reacted with Rapid PNGase-F (New England Biolabs) as described elsewhere^36^. Liberated glycosylamines were reacted with RapiFluor-MS, then desalted on PD Minitrap G-10 columns. RapiFluor-MS-labelled glycans from 8 reactions were pooled and dried in a centrifugal evaporator for mass spectrometry analysis.

### ESI-MS^2^ of RapiFluor-MS-labelled N-glycans

Labelled glycans were reconstituted in 40 µl of a mixed solvent composed of 90:100:210 Water:Dimethylformamide:Acetonitrile. 10 µl injections were separated on an Agilent 1290 UPLC using the same column, flow rate and mobile phase conditions as described in the paragraph *Characterization of Labelled Glycan Standards’ Purity and Concentration*. The Agilent 1290 UPLC was connected in tandem to an Agilent 6550 iFunnel qToF. The following parameters were optimized for ESI-MS: drying gas and sheath gas flowrate 12 L/min, nebulizer pressure 45 psig, drying gas temperature 150 °C, sheath gas temperature 300 °C, capillary voltage 2500 V, fragmentor voltage 150 V. The RF voltage of high pressure and low pressure ion funnels were 90 and 40 V respectively. The MS was operated in positive mode over a mass range of m/z 300–2000 with an acquisition time of 1 sec per spectrum. Mass correction was enabled using an infused calibrant solution with a reference mass of 922.0098 over a detection window of 50ppm.

Precursor ions eluting at the relevant normalized retention times indicated by shotgun glycan microarray experiments and determined by ExPASy GlycoMod^68^ to be fucosylated N-glycans were chosen for MS^2^. Targeted MS/MS was performed for the fucosylated N-glycans in positive mode over a mass range of m/z 100–2000 with an acquisition time of 1.5 sec per spectrum. The collision energy used for the tandem experiment was calculated based on m/z value using the relationship of CE (eV) = 1.5*(m/z)/100−4.8. All data was processed in Agilent MassHunter software.

### Data Availability Statement

The datasets generated during the current study are available from the corresponding author on reasonable request.

## Electronic supplementary material

Supplementary Information

mAb A4 chemically defined glycan microarray

mAb A4 shotgun glycan microarray
