# Supplementary material for: Shotgun Glycomics Identifies Tumor-Associated Glycan Ligands Bound by an Ovarian Carcinoma-Specific Monoclonal Antibody
Source: Sci Rep. 2017 Nov 3;7:14489. doi: 10.1038/s41598-017-15123-z (PMC5670200; doi:10.1038/s41598-017-15123-z)
Supplement: Supplementary file 1 — Supplementary Information [file 41598_2017_15123_MOESM1_ESM.pdf]

Shotgun Glycomics Identifies Tumor-Associated Glycan Ligands Bound by an Ovarian Carcinoma-Specific Monoclonal Antibody

Liau B, Tan B, Teo G, Zhang P, Choo A, Rudd P.M.

High-resolution scans of representative microarray slides as well as their corresponding .gal files are provided in the zipped archives “mAb A4 Chemically Defined.zip” and “mAb A4 Shotgun.zip”.

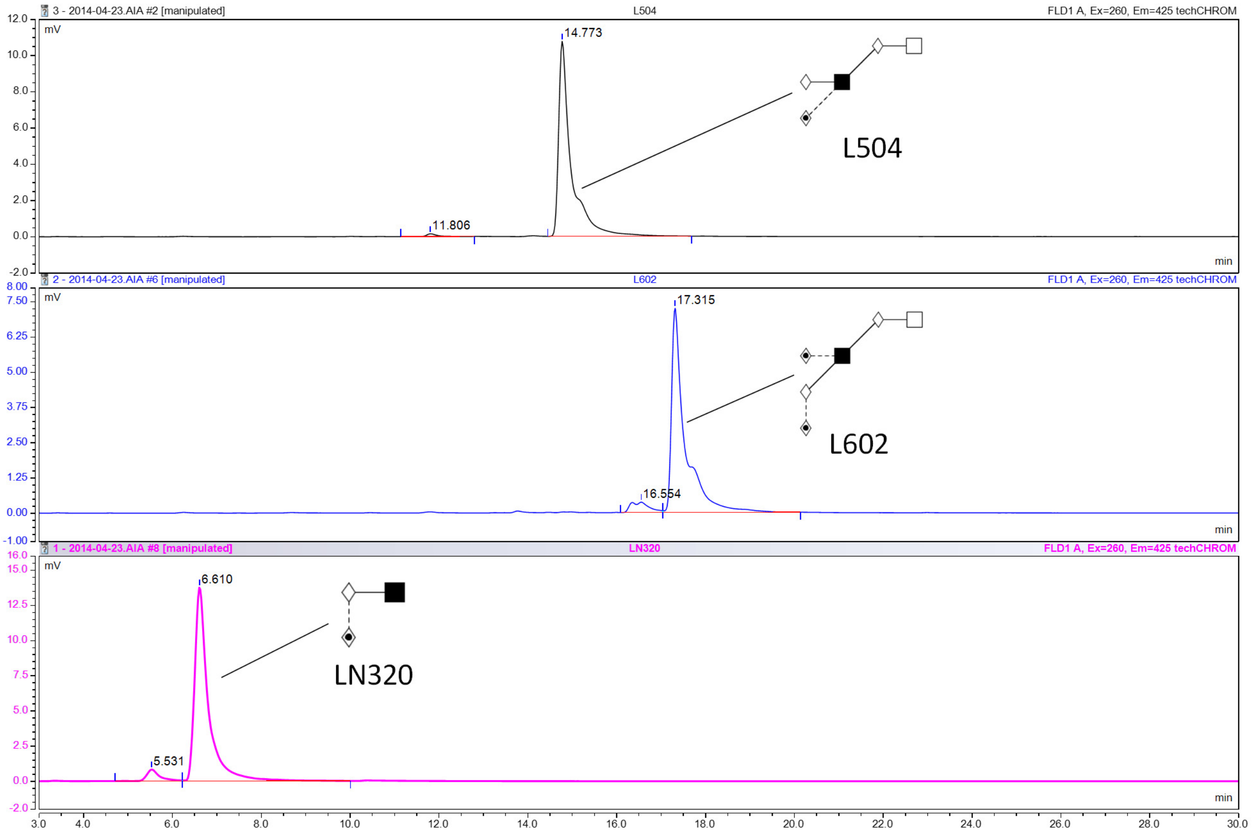

Supplementary Fig. 1. HILIC-UPLC characterization of AEAB-labelled glycan standards. HILIC-UPLC with fluorescence detector was used to characterize the purity and concentration of AEAB-labelled glycan standards used for chemically defined glycan array.

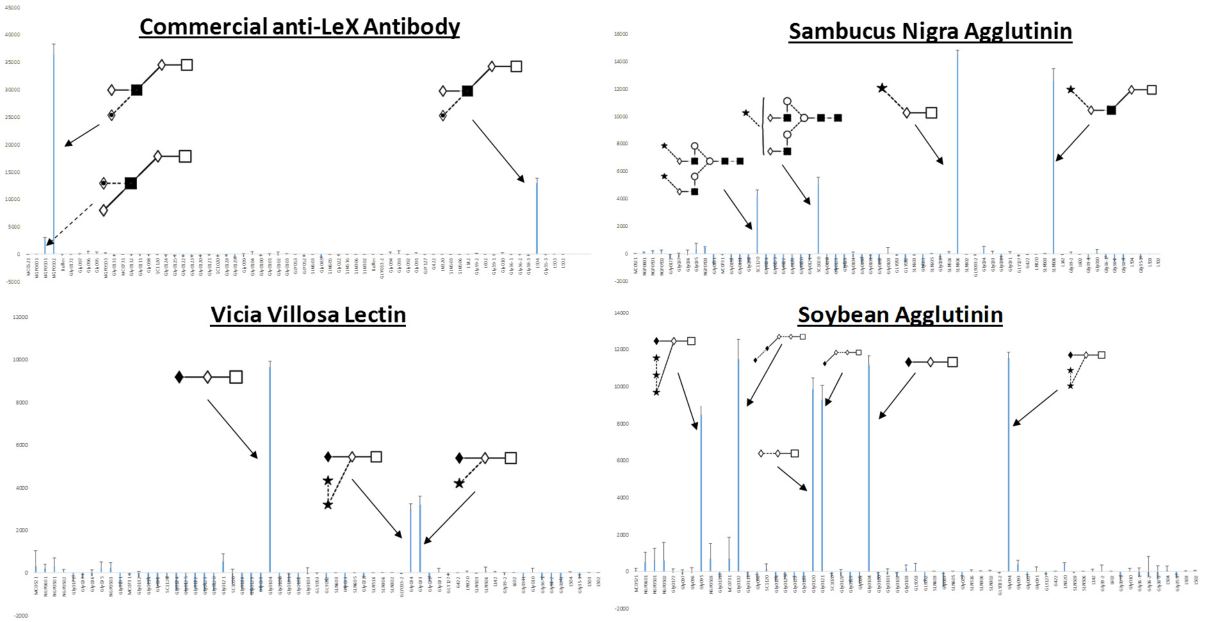

Supplementary Fig. 2. Validation of chemically defined glycan array using plant lectins and commercial antibodies. Good signal-to-noise ratios were observed using standard lectins and antibodies of known binding specificity. Results were in good agreement with publicly available results in the Consortium For Functional Glycomics database.

A.

Monosaccharide	Colour	Black / White
Glucose	□	□
Galactose	◇	◇
N-acetyl Glucosamine	■	■
N-acetyl Galactosamine	◆	◆
Mannose	○	○
Fucose	◈	◈
N-acetyl Neuraminic Acid	★	★

B.

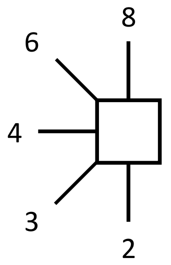

C.

—— = β linkage

------ = α linkage

Supplementary Fig. 3. Oxford notation for the representation of glycan structures.

Minimising band broadening effect	Determination of dwell volume
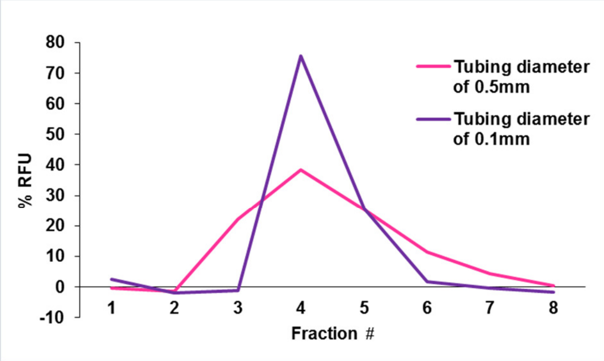	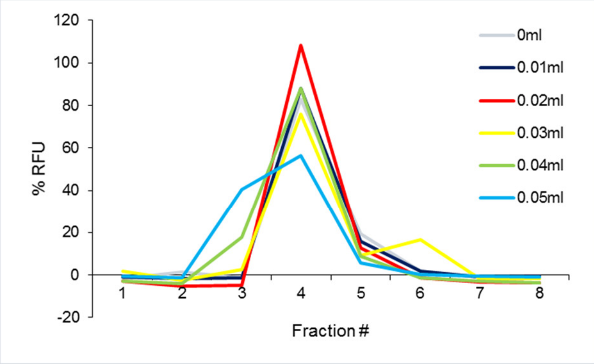
✓ Tubing diameter of 0.1mm	✓ Dwell volume of 0.02ml

#	Start (min)	End (min)	Condition	% Peak collection
1	2.02	2.20	Time	100
2	2.22	2.40	Time	100
3	2.42	2.60	Time	100
4	2.62	2.80	Time	100
5	2.82	3.00	Time	100
6	3.02	3.20	Time	100
7	3.22	3.40	Time	100
8	3.42	3.60	Time	100

Supplementary Fig. 4. Optimization of Waters Fraction Collector III. Fractions were collected at 0.2min intervals after injection of a lactose-AEAB standard. Band broadening was minimized through the use of narrower tubing. Setting the instrument to the correct dwell volume of 0.02ml resulted in the highest percentage of intensity in fraction 4.

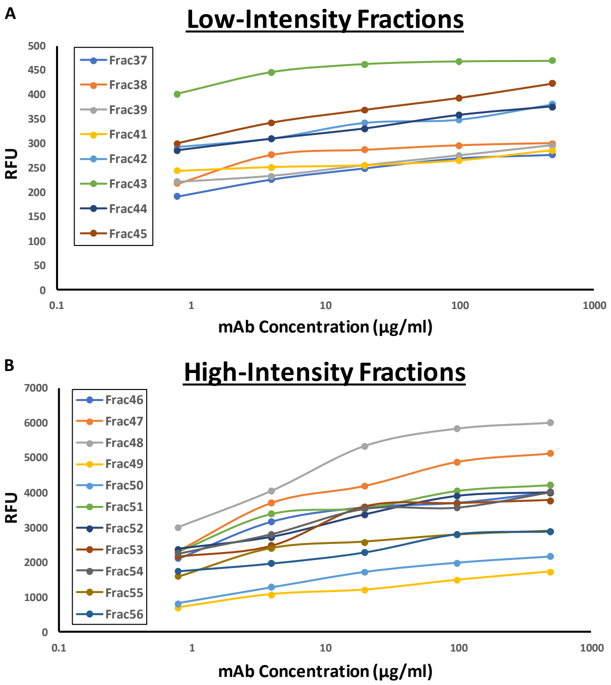

Supplentary Fig. 5. Changes in mAb A4 binding signal to shotgun glycan microarray over a range of antibody concentrations. Fluorescent signals from (A) low-intensity and (B) high-intensity positive fractions were measured at mAb A4 concentrations of 0.8, 4, 20, 100 and 500µg/ml. 20µg/ml was chosen as an appropriate concentration for binding inhibition experiments because it showed favorable signal intensity with minimal noise, and was on the upper end of the binding curve for both low and high-intensity fractions.

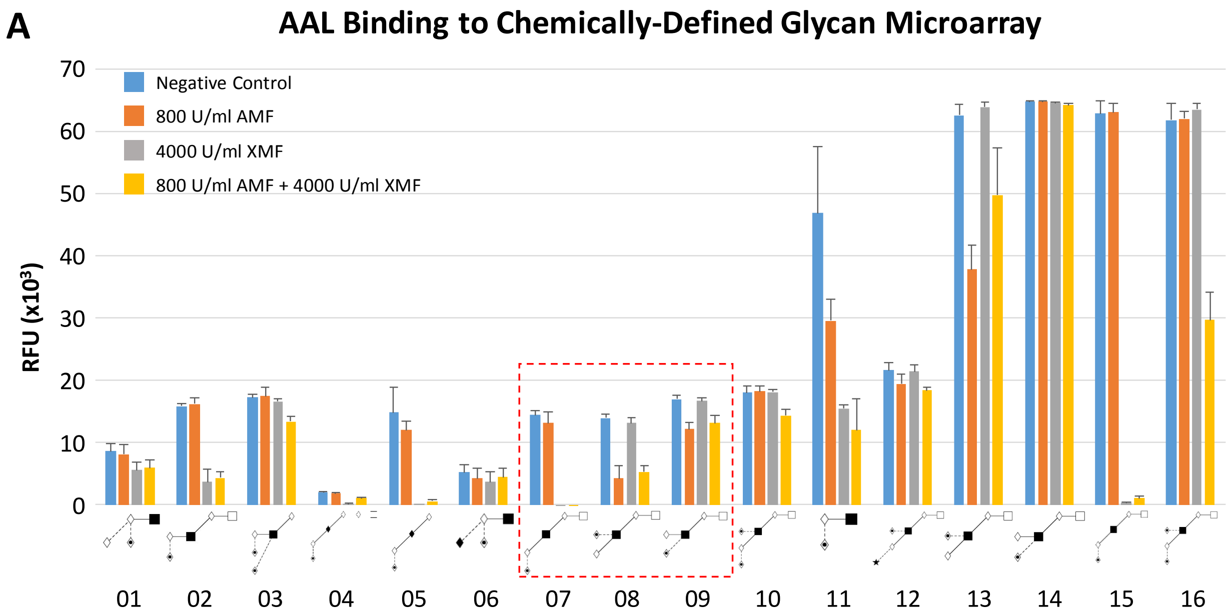

Supplementary Fig. 6. Optimization of XMF and AMF exoglycosidases on chemically defined glycan array. On-slide exoglycosidase digestions were shown to be efficacious by measuring the decrease in AAL binding intensity to chemically defined glycan array. Of particular note are the isomeric structures highlighted in the dashed red box – XMF digestion is completely effective at removing terminal α1-2 fucose, but AMF is only partially effective at removing α1-3 and less effective at removing α1-4 fucose. XMF is less effective with shorter glycan structures such as structure 11.

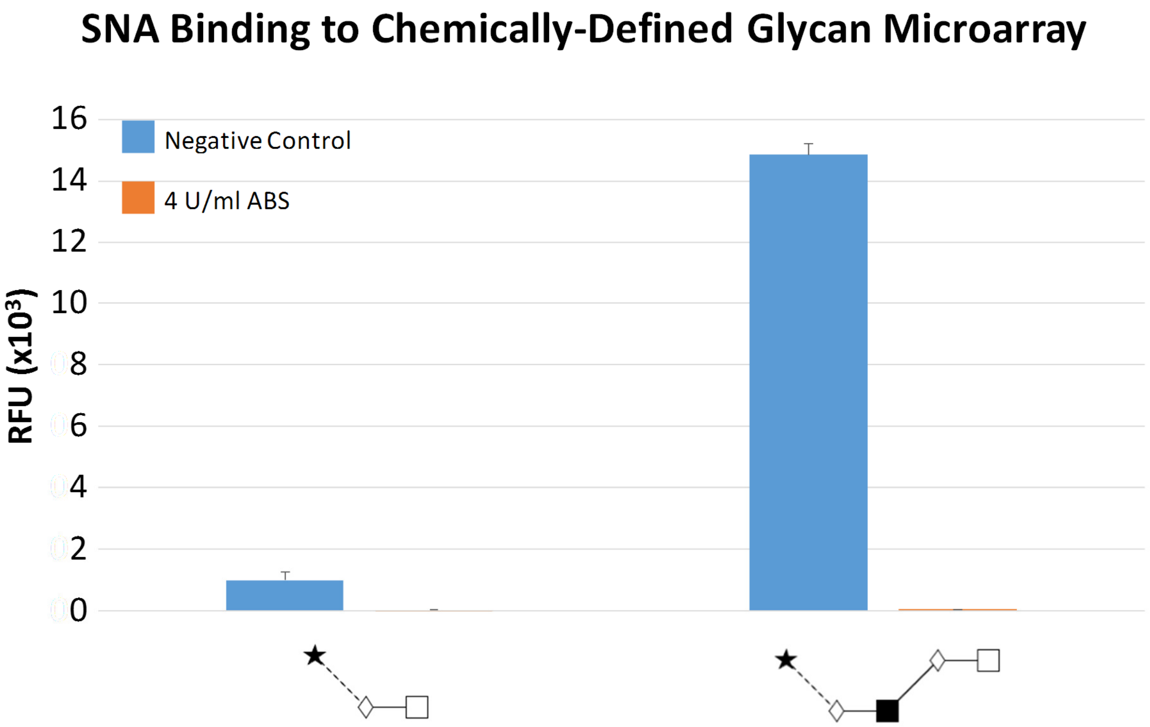

Supplementary Fig. 7. Optimization of ABS exoglycosidase on chemically defined glycan array. SNA lectin binding was completely abolished by treatment with ABS.

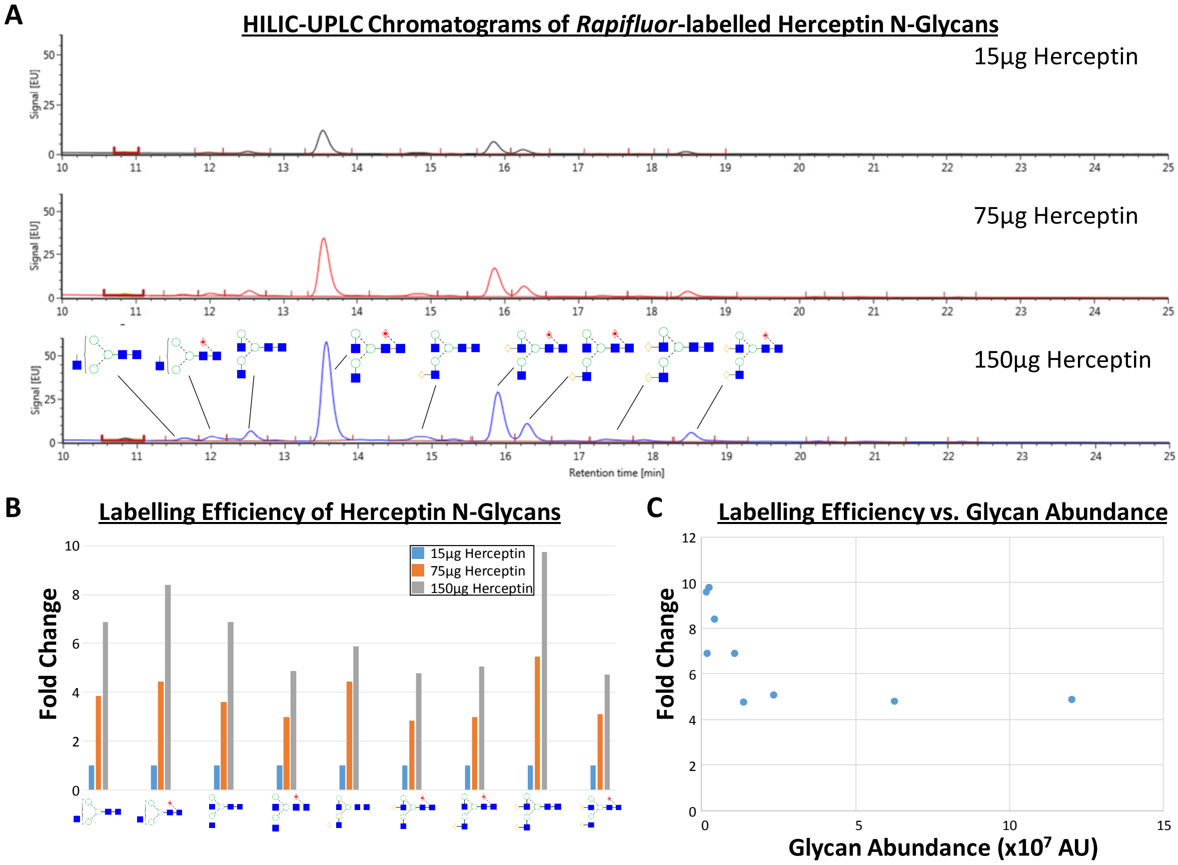

Supplementary Fig. 8. Optimization of RapiFluor-MS reaction with quantities of glycoprotein greater than recommended by manufacturer. (A) HILIC-UPLC traces of N-glycans released from 15, 75 and 150µg of Herceptin monoclonal antibody, with annotated structures. (B) Quantification of variation in labelling efficiency of each glycan structure. Although the fold-increase in signal intensity was systematically less than the fold-increase in mass of protein, there was no bias related to glycan structure. (C) Quantification of variation in labelling efficiency of glycans in relation to their relative abundance. Low abundance glycans were labelled with higher efficiency than highly abundant glycans. Although this is potentially problematic for glycan quantification experiments, this bias was not expected to be problematic for this study since we were interested in structural characterization of low abundance structures.

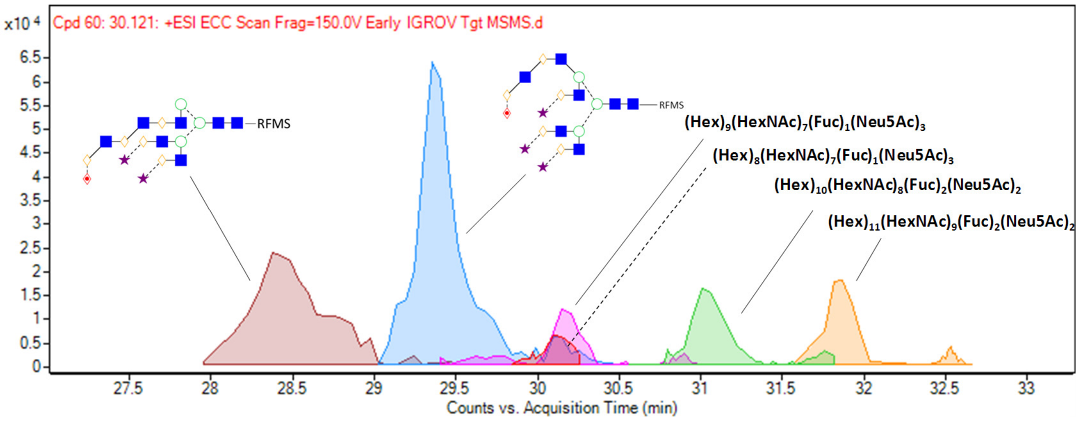

Supplementary Fig. 9. Extracted ion chromatogram of Rapifluor labelled IGROV-1 complex N-glycans. Signal intensities of late-eluting N-glycans (> 28 min) were progressively smaller and smaller. Two structures were of sufficient abundance to permit structural characterization using MS/MS.

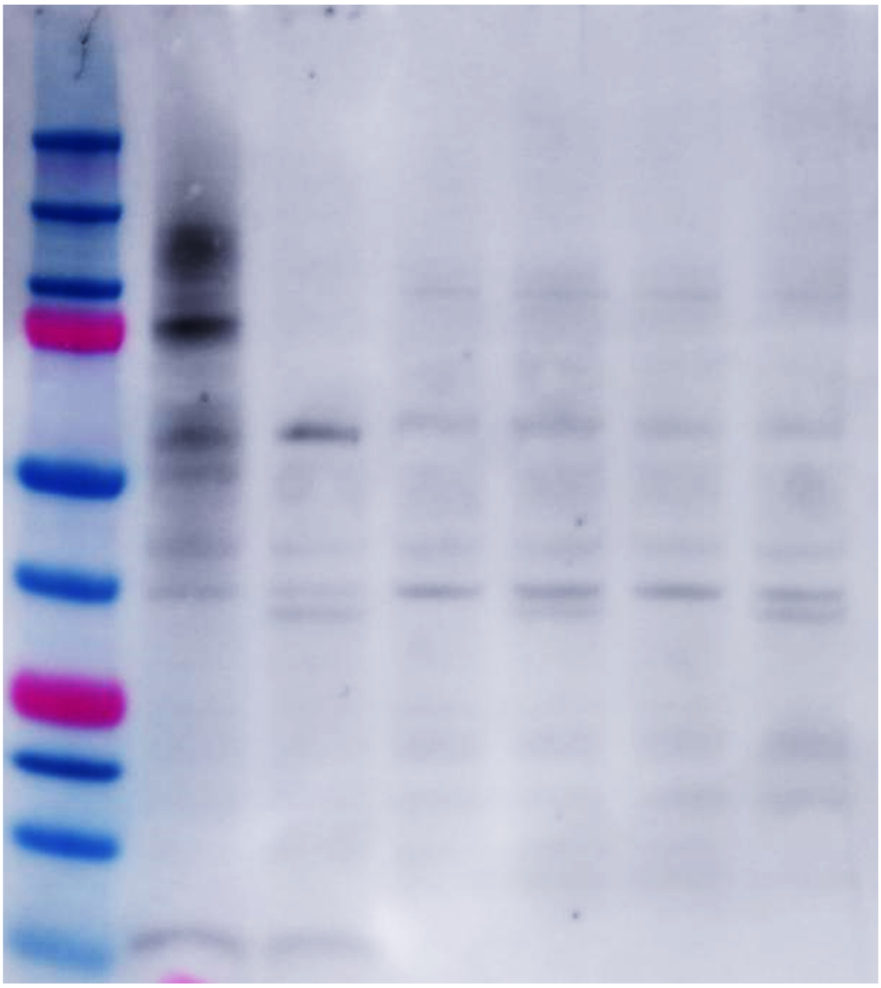

Supplementary Fig. 10. Full-length Western Blot of IGROV-1 and IOSE-523 cell lysates. Uncropped blot shown in Fig. 1B. Lane 1: Molecular weight marker (Bio-Rad Precision Plus Protein Dual Color Standards), Lane 2: IGROV-1 cell lysate, Lane 3: IGROV-1 cell lysate treated with PNGase-F, Lane 4: IOSE523 cell lysate, Lane 5: IOSE523 cell lysate treated with PNGase-F, Lane 6: IOSE523 cell lysate repeat, Lane 7: IOSE523 cell lysate treated with PNGase-F repeat.

Supplementary Table 1

Glycan ID	Structure	Purity (%)
L502	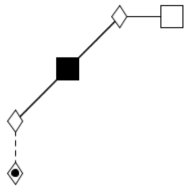	100
L504	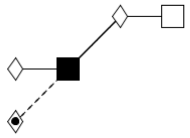	99.0
L503	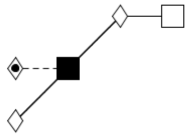	97.2
L602	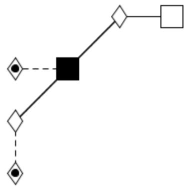	93.4
LN320	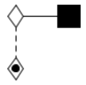	94.6
SLN302	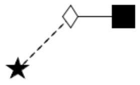	94.6
SLN506	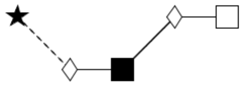	86.9
SLN605	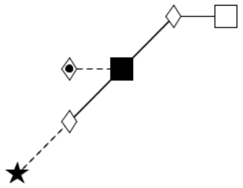	56.3
G422	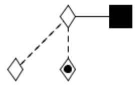	97.8

L142	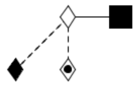	96.6
SLN516	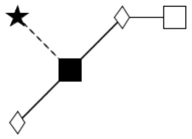	82.0
SLN603	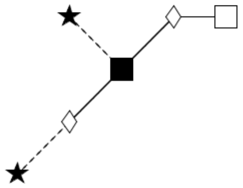	95.9
SLN306	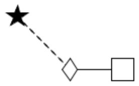	100
SLN503	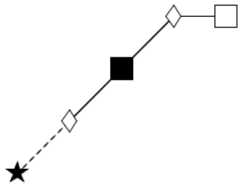	58.1
GLY33-2	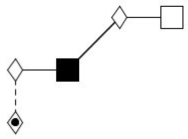	100
GLY53	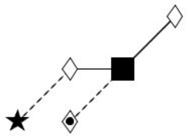	92.9
GLY127	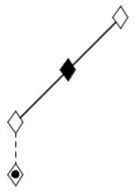	91.1

GLY52	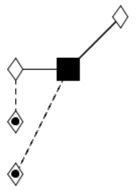	100
Gly10	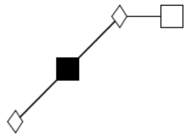	100
Gly22	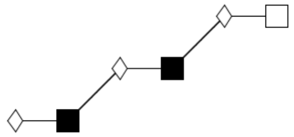	100
Gly35-3	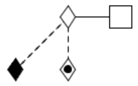	100
Gly36-1	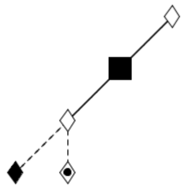	100
Gly36-2	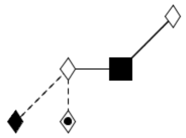	53.5
Gly38-3	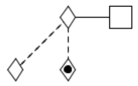	100
Gly39-1	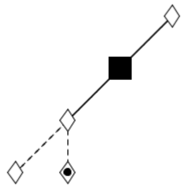	100
Gly39-2	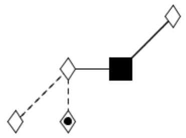	85.5

Gly83	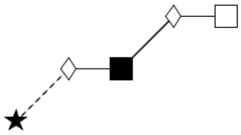	100
Gly91	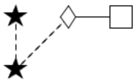	100
Gly92	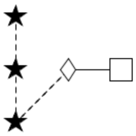	82.7
Gly93	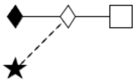	100
Gly94	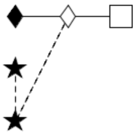	100
Gly95	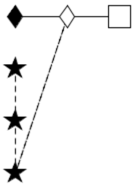	100
Gly96	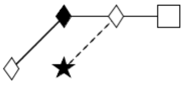	100
Gly97	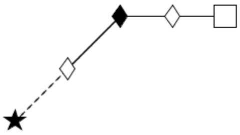	100
Gly98	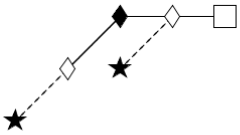	94.92957746

Gly99	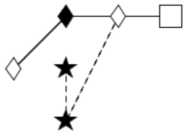	100
Gly100	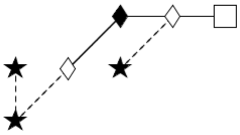	100
Gly101	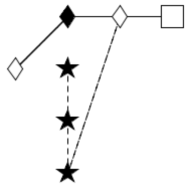	100
Gly102	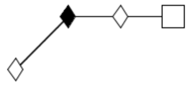	93.93268379
Gly103	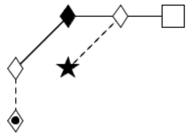	100
Gly104	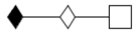	100
Gly111	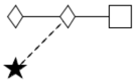	87.25361367
Gly120	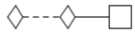	100
Gly121	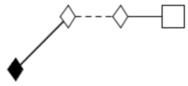	88.7678693
Gly122	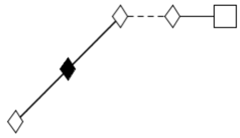	92.12244898

Gly123	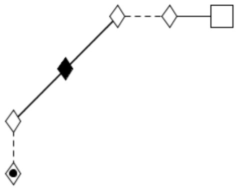	90.2359642
Gly124	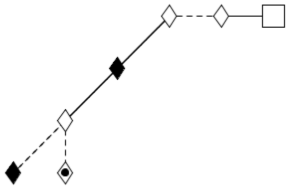	88.7628866
Gly125	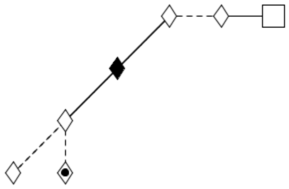	94.15292354
Gly128	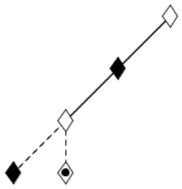	100
Gly129	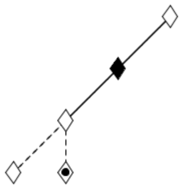	100
Gly131	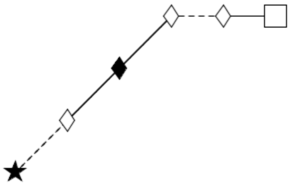	93.85159011
Gly132	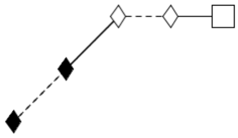	94.48979592

Gly172	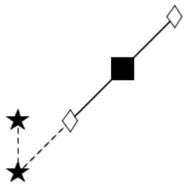	100
SC1020	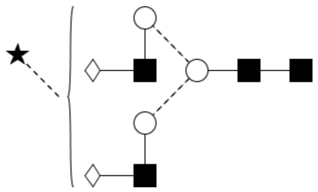	83.8
SC1120	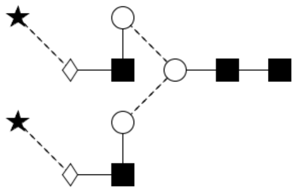	81.8
MC0521	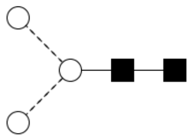	90.1
MC0731	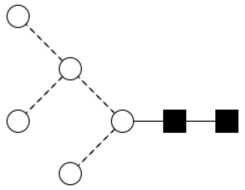	87.6
NGP0501	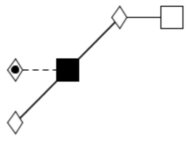	N/D
NGP0502	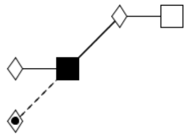	N/D

NGP0503	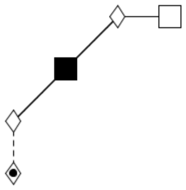	N/D
NGP0601	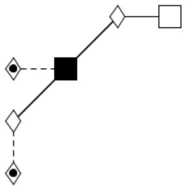	N/D
