# Supplementary figures and images for: Shotgun Glycomics Identifies Tumor-Associated Glycan Ligands Bound by an Ovarian Carcinoma-Specific Monoclonal Antibody
Source: Sci Rep. 2017 Nov 3;7:14489. doi: 10.1038/s41598-017-15123-z (PMC5670200; doi:10.1038/s41598-017-15123-z)

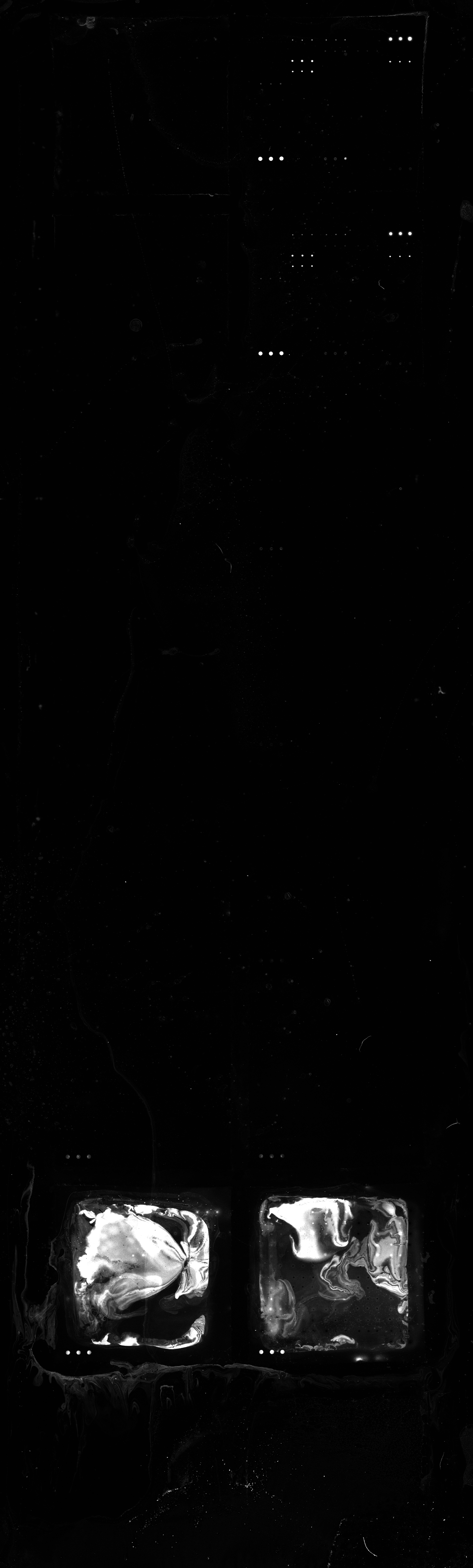

Supplement: Supplementary file 2 — mAb A4 chemically defined glycan microarray [file 41598_2017_15123_MOESM2_ESM.tif]

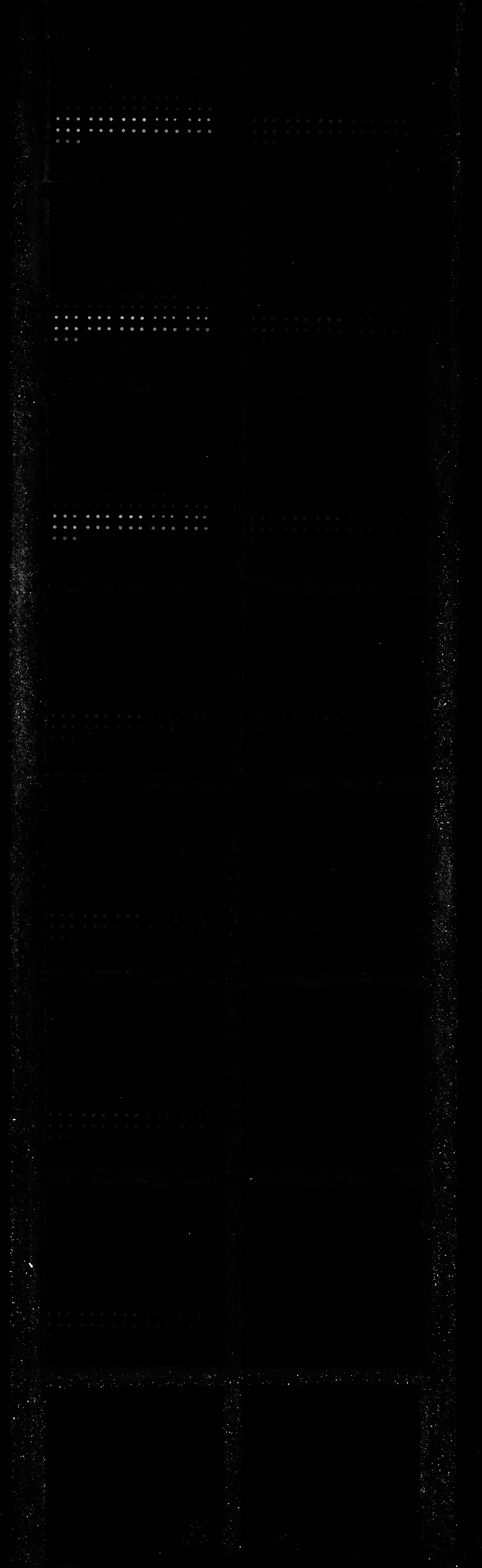

Supplement: Supplementary file 3 — mAb A4 shotgun glycan microarray [file 41598_2017_15123_MOESM3_ESM.tif]
